# Cost-Effectiveness of Risk-Reducing Surgery for Breast and Ovarian Cancer Prevention: A Systematic Review

**DOI:** 10.3390/cancers14246117

**Published:** 2022-12-12

**Authors:** Xia Wei, Samuel Oxley, Michail Sideris, Ashwin Kalra, Li Sun, Li Yang, Rosa Legood, Ranjit Manchanda

**Affiliations:** 1 Department of Health Services Research and Policy, London School of Hygiene & Tropical Medicine, London WC1H 9SH, UK; 2 Wolfson Institute of Population Health, CRUK Barts Cancer Centre, Queen Mary University of London, London EC1M 6BQ, UK; 3 Department of Gynaecological Oncology, Barts Health NHS Trust, Royal London Hospital, London E1 1BB, UK; 4 School of Public Health, Peking University, Beijing 100191, China; 5 MRC Clinical Trials Unit at UCL, Institute of Clinical Trials & Methodology, Faculty of Population Health Sciences, University College London, London WC1V 6LJ, UK; 6 Department of Gynaecology, All India Institute of Medical Sciences, New Delhi 110029, India

**Keywords:** risk-reducing surgery, breast cancer, ovarian cancer, BRCA, cost-effectiveness

## Abstract

**Simple Summary:**

Synthesised cost-effectiveness evidence is necessary for resource allocation for risk-reducing surgery in breast cancer (BC)/ovarian cancer (OC)/endometrial cancer (EC) prevention strategies. We aimed to review evidence on the cost-effectiveness of surgical prevention for BC/OC/EC in high/intermediate/low-risk populations. From 22 included studies, risk-reducing mastectomy (RRM) and/or risk-reducing salpingo-oophorectomy (RRSO) were cost-effective for *BRCA1/2*, and RRSO was also cost-effective at a lower lifetime OC risk-threshold of 4–5%. Risk-reducing early salpingectomy and delayed oophorectomy (RRESDO) was cost-effective compared to RRSO in one-study. Hysterectomy with bilateral salpingo-oophorectomy (BSO) was cost-effective in Lynch syndrome women. Opportunistic bilateral salpingectomy (OBS) was cost-effective when conducted with hysterectomy for benign gynaecology surgery or in lieu of tubal sterilisation. This systematic review found that surgical prevention is cost-effective for women who are at high risk of BC, intermediate/high risk of OC and high risk of EC. The results are sensitive to age, disutility and uptake rates regarding RRS along with the effect sizes in terms of OC-risk reduction from salpingectomy. These areas require further research. **Key points**: **Question:** What is the evidence on the cost-effectiveness of risk-reducing surgery for breast, ovarian and endometrial cancer prevention? **Findings:** This systematic review found that surgical prevention is cost-effective for women in high-income countries who are at a high risk of breast cancer, intermediate/high risk of ovarian cancer and high risk of endometrial cancer. The results, while robust for most strategies, are sensitive to certain parameters, especially the disutility from surgery and the effect sizes of ovarian cancer risk reduction from salpingectomy. **Implications:** Risk-reducing surgery is cost-effective for breast/ovarian/endometrial cancer prevention across most settings, but more research is needed on the disutility from all preventive surgery and the precision in terms of the cancer risk reduction from salpingectomy.

**Abstract:**

Policymakers require robust cost-effectiveness evidence of risk-reducing-surgery (RRS) for decision making on resource allocation for breast cancer (BC)/ovarian cancer (OC)/endometrial cancer (EC) prevention. We aimed to summarise published data on the cost-effectiveness of risk-reducing mastectomy (RRM)/risk-reducing salpingo-oophorectomy (RRSO)/risk-reducing early salpingectomy and delayed oophorectomy (RRESDO) for BC/OC prevention in intermediate/high-risk populations; hysterectomy and bilateral salpingo-oophorectomy (BSO) in Lynch syndrome women; and opportunistic bilateral salpingectomy (OBS) for OC prevention in baseline-risk populations. Major databases were searched until December 2021 following a prospective protocol (PROSPERO-CRD42022338008). Data were qualitatively synthesised following a PICO framework. Twenty two studies were included, with a reporting quality varying from 53.6% to 82.1% of the items scored in the CHEERS checklist. The incremental cost-effectiveness ratio/incremental cost-utility ratio and cost thresholds were inflated and converted to US$2020, using the original currency consumer price index (CPI) and purchasing power parities (PPP), for comparison. Eight studies concluded that RRM and/or RRSO were cost-effective compared to surveillance/no surgery for *BRCA1/2*, while RRESDO was cost-effective compared to RRSO in one study. Three studies found that hysterectomy with BSO was cost-effective compared to surveillance in Lynch syndrome women. Two studies showed that RRSO was also cost-effective at ≥4%/≥5% lifetime OC risk for pre-/post-menopausal women, respectively. Seven studies demonstrated the cost-effectiveness of OBS at hysterectomy (n = 4), laparoscopic sterilisation (n = 4) or caesarean section (n = 2). This systematic review confirms that RRS is cost-effective, while the results are context-specific, given the diversity in the target populations, health systems and model assumptions, and sensitive to the disutility, age and uptake rates associated with RRS. Additionally, RRESDO/OBS were sensitive to the uncertainty concerning the effect sizes in terms of the OC-risk reduction and long-term health impact. Our findings are relevant for policymakers/service providers and the design of future research studies.

## 1. Introduction

Breast cancer (BC) is the most frequent female malignancy, affecting 2.26 million women per year [1]. Ovarian cancer (OC) affects 314,000 women annually and is the most lethal of all gynaecological cancers due to its poor biology and advanced stage at presentation [1,2]. As a result, these two cancers lead to substantial medical and economic burdens for both patients as individuals and the healthcare system as a whole [3,4]. Around 4% of BC [5,6] and 15–20% of OC cases [7,8] and 3% of endometrial cancer (EC) cases [9] are caused by known pathogenic variants (PVs) in a variety of cancer susceptibility genes (CSGs); many of these CSGs can cause both BC and OC or EC and OC. Women who carry a PV in one of these CSGs or have a strong family-history (FH) are at increased cancer-risk compared to the general population of women. The majority of these CSG-associated cancers are potentially preventable.

A number of prevention strategies have been used to reduce BC/OC incidence and mortality. Risk-reducing surgery (RRS) is considered to be the most effective preventive strategy for unaffected women at elevated-risk of BC/OC. Risk-reducing mastectomy (RRM) provides a BC risk reduction of 89–95% [10,11,12] and is commonly offered to women with an estimated lifetime BC risk over 30–40% [13,14]. Similarly, risk-reducing salpingo-oophorectomy (RRSO) is the gold-standard preventive strategy for OC [15]. RRSO provides a risk reduction of up to 97% [15,16,17]. While it has previously been offered to women with an estimated ≥10% lifetime OC risk, it is now recommended at >4–5% lifetime OC-risk, including women with moderate-penetrance CSGs [18,19,20]. In women with Lynch syndrome (LS), hysterectomy with bilateral salpingo-oophorectomy (BSO) is the recommended preventative operation, as, apart from OC, such women also have an increased-risk of EC [21]. Recently, the widespread acceptance of the fallopian tube as the origin of most serous epithelial OC has supported a novel approach for OC risk reduction in high-risk women which includes risk-reducing early-salpingectomy (RRES) followed by delayed oophorectomy (DO) (RRESDO). Ongoing trials [22,23,24] are evaluating relevant outcomes including sexual function, menopause, quality of life and oncological prevention. In a similar context, opportunistic bilateral salpingectomy (OBS) during benign gynaecological surgery in baseline risk general population women provides a level of OC risk-reduction estimated to range from 28% to 65% and is being recommended in clinical practice [25,26,27].

Due to financial pressures in health systems, policymakers need to make difficult decisions regarding healthcare resource allocation, mainly based on the effectiveness and cost-effectiveness of interventions. Economic evaluation is thus critically important to weigh up the costs and consequences of alternative health strategies to help health policy decision-making. For interventions to be sustainable, they need to be cost-effective and affordable. The clinical effectiveness of RRM/RRSO is well-established, and studies have demonstrated that RRM/RRSO are cost-effective compared to surveillance or no intervention for *BRCA1/2* PV carriers [28,29]. Earlier efforts to synthesise available evidence on the cost-effectiveness of RRS are limited and have been restricted to RRM/RRSO for *BRCA* PV carriers [30] or limited to data from western health systems [31]. However, more recent studies demonstrate RRSO to be cost-effective for pre- and post-menopausal women at lower lifetime OC risk levels of 4–5% [19,20], which can save 7–10 years of a woman’s life. For women with LS, earlier studies have reported that hysterectomy with BSO were cost-effective to prevent OC and EC relative to no surgery [32,33]. Additionally, a number of recent studies indicate that RRESDO in high-risk women and OBS in baseline-risk women undergoing pelvic surgery could be cost-effective strategies for decreasing OC-risk [34,35,36].

The aim of this systematic review was to critically evaluate and summarise the published evidence and identify knowledge gaps on the cost-effectiveness (and related methodology) of RRS including: RRM for the prevention of BC, RRSO/RRESDO/OBS for the prevention of OC and hysterectomy for the prevention of EC in LS women. The results of this review can facilitate the decision making of healthcare policymakers regarding health resource allocation as well as inform the design of future studies to address knowledge gaps for economic analysis on this topic.

## 2. Materials and Methods

This systematic review was based on a prospectively registered protocol (PROSPERO: CRD42022338008) in line with PRISMA (Preferred Reporting Items for Systematic reviews and Meta-Analyses).

### 2.1. Literature Search

We searched major databases (Medline, Embase, PubMed and the Cochrane Library) from inception to December 2021 using a predefined search strategy (Supplementary File S1). Searches were limited to English titles and human studies. Additionally, we manually searched reference lists of relevant primary studies or review articles.

### 2.2. Study Selection (Inclusion Criteria)

We defined our inclusion criteria based on the “Population, Intervention, Comparator, Outcomes—(PICO)” framework; we used two distinct frameworks to formulate our methods. Our first PICO framework included a population of women who are at high risk of BC or at increased risk of OC as defined by the diagnosis of PV in a CSG (e.g., *BRCA1/2* and *PALB2* for BC; or *BRCA1/2, RAD51C*, *RAD51D*, *BRIP1* and *PALB2* for OC) or by the diagnosis of LS or by a strong FH of BC and/or OC. We defined the intervention as RRSO/RRESDO in the case of OC prevention and RRM for BC prevention, along with hysterectomy and BSO in the case of EC and OC prevention for LS. Our comparators included any non-surgical alternative, i.e., no action (conservative approach)/surveillance/chemoprevention. We focused on modelling or trial-based economic evaluation studies which reported on cost-effectiveness or cost-utility outcomes.

Our second PICO framework included women at baseline (general population level) OC-risk who underwent OBS either as a form of sterilisation (including at caesarean section) or at a gynaecological surgery (e.g., hysterectomy). Outcomes were as above (first PICO) and were compared with the standard surgical approach without salpingectomy (e.g., tubal ligation/hysterectomy alone).

### 2.3. Screening of the Literature

**Figure 1:**
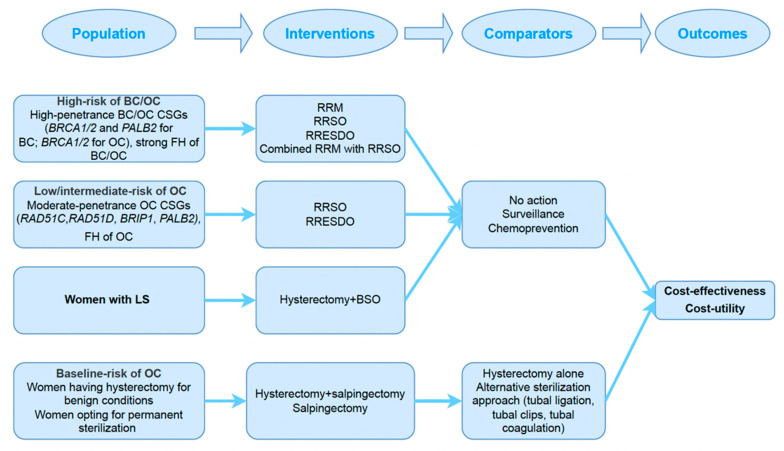
Systematic review protocol. BC, breast cancer; BSO, bilateral salpingo-oophorectomy; CSGs, cancer susceptibility genes; FH, family history; LS, Lynch syndrome; OC, ovarian cancer; RRESDO, risk-reducing early salpingectomy with delayed oophorectomy; RRM, risk-reducing mastectomy; RRSO, risk-reducing salpingo-oophorectomy.

Retrieved titles were initially transferred into reference management software (EndNote 20.2, Clarivate Analytics) and duplicates were removed. Titles and abstracts were screened by two independent reviewers (XW and SO). Eligible full texts of the shortlisted abstracts were assessed for inclusion. Any disagreement was resolved by a third reviewer (MS) or the senior author of the study (RM). We included economic evaluation studies that followed our PICO frameworks (Figure 1) and reported on cost-effectiveness or cost-utility outcomes.

### 2.4. Exclusion Criteria

We excluded studies that (i) reportedly included women who underwent RRM with a personal history of BC or RRSO/RRESDO/OBS with a personal history of OC; (ii) only reported either costs or clinical efficacy; (iii) conducted cost minimisation analysis; (iv) only conducted budget impact analysis; and (v) were case reports or (vi) review articles.

### 2.5. Data Extraction

Data were extracted by XW using predesigned Microsoft Excel spreadsheet tables and cross-checked by SO with any disagreements resolved by MS/RM. The extracted data included settings, year of study, population parameters (risk classification, gene or FH-based diagnosis), surgical intervention and the reported economic evaluation outcomes for each population.

We also reviewed methodological characteristics including model assumptions, types of economic evaluation, perspective of analysis, study design, time horizon, sources and the reporting of costs and effectiveness, outcome measures, discount, incremental analysis, and types of sensitivity analysis. The types of economic evaluation were categorised as cost-effectiveness analysis (CEA) and cost-utility analysis (CUA). The outcome measures included cancer cases/death prevented, life year gained (LYG) and quality-adjusted life year (QALY). Included studies may have been conducted from a payer or societal perspective. Study designs included trial-based, observational studies-based (cross-sectional, cohort, case control), decision tree model, Markov model, and microsimulation model. We classified the sources/reporting of costs and effectiveness as primary data (e.g., questionnaires from individual patients) and secondary data (e.g., unit cost lists), published literature and expert opinion. We also explored sensitivity analyses and categorised them in one-way or probabilistic sensitivity analysis (PSA).

### 2.6. Quality of Reporting

We used the most updated Consolidated Health Economic Evaluation Reporting Standards 2022 (CHEERS 2022) to assess whether the reported health economic evaluations were identifiable, interpretable and could act as a useful adjunct for decision making [37]. Two independent reviewers (XW/SO) scored the 28 items with 0 (not reported), 0.5 (not fully reported), 1 (fully reported) or NA (not applicable) depending on the reporting quality of each study. We added up all the items’ scores to calculate an overall score for each study. The ratio of this score divided by the maximum obtainable score provides a % which reflects the reporting quality for each study.

### 2.7. Evidence Synthesis

Given heterogeneity across populations (country, healthcare systems) and willingness-to-pay (WTP) thresholds, we did not undertake a meta-analysis. Instead, we performed a qualitative synthesis of the included data and presented them in comprehensive tables based on our PICO frameworks. To further facilitate comparison across studies, the ICER/ICUR and costs threshold from the sensitivity analysis reported in each study were inflated and converted to US$2020, using the consumer price index (CPI) [38] of the original currency and purchasing power parities (PPP) [39]. Due to the variation in WTP thresholds in different countries, we did not use a specific WTP threshold, and the cost-effectiveness of RRS was evaluated based on the country-specific WTP threshold used in each study.

### 2.8. Accuracy of Data

XW and RM are guarantors of the data quality. The corresponding and senior author (RM) made the final decision to submit for publication.

## 3. Results

### 3.1. Study Characteristics

**Figure 2:**
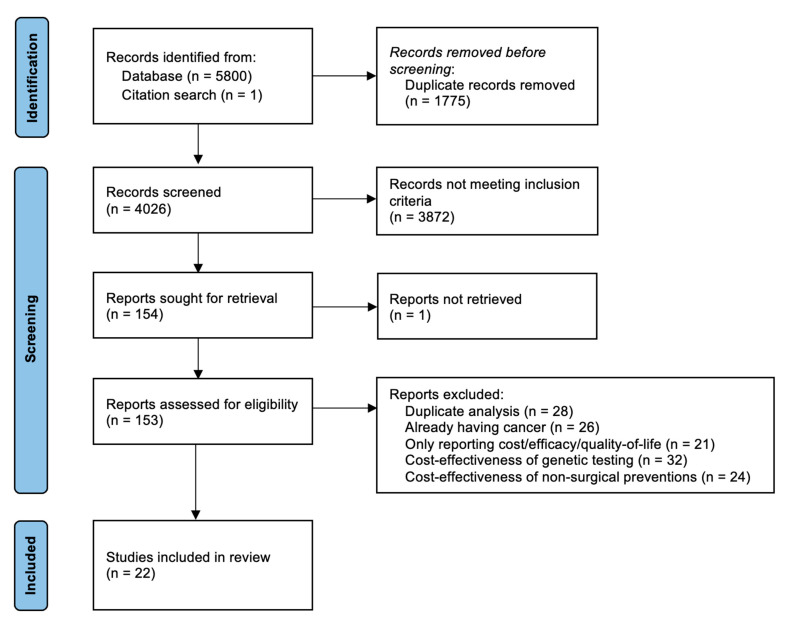
PRISMA flow diagram.

Our database search yielded 5800 records, and one additional record was added from a reference check for two systematic reviews [30,31]. Twenty-three studies were eligible for inclusion. However, one study’s full text [40] could not be retrieved. Hence, 22 studies were included in our qualitative synthesis. Figure 2 summarises the study selection process. The included studies were published between 1998 and 2021 and came from eight countries (N = twelve from USA, N = three from Canada, N = two from UK, N = one each from Australia, Germany, Japan, Norway, Switzerland). Ten studies included women at high-risk of BC/OC. Amongst them, nine studies included unaffected women with *BRCA1/2* PV (eight studies for RRM or RRSO or combined RRM and RRSO, one for RRESDO) and one study included RRM for *BRCA1/2* PV carriers affected by OC. Three studies included women with LS who underwent hysterectomy and BSO. Two studies included women who were at low/intermediate risk of OC and underwent RRSO. Seven studies included women at baseline population risk of OC who underwent OBS as part of surgery for a benign gynaecological condition or sterilisation. Supplementary File S2 summarises the characteristics of the included studies, and important assumptions and limitations of the included studies.

### 3.2. Reported Methodology of Included Studies

Table 1 summarises a breakdown of the economic evaluation methodologies used in the 22 included studies. Four studies performed CEA, reporting the number of cancer cases/deaths prevented or LYG as outcome measures, whereas seven studies conducted CUA and reported QALY as outcome measures. Eleven studies conducted both CUA/CEA. Nine studies adopted the payer perspective, and nine studies adopted the societal perspective, with four studies not specifying a study perspective. Regarding study design, 14 studies developed a Markov model with a lifetime horizon. Seven studies used a decision tree with a time horizon from five years to lifetime, and one study conducted microsimulation with a lifetime horizon. Twenty studies performed incremental analysis and reported ICER/ICUR, whereas two studies only reported the average cost-effectiveness. Nine studies conducted deterministic sensitivity analysis (one-way/two-way) to explore the uncertainty, whereas 13 studies conducted both deterministic sensitivity analysis and PSA.

Supplementary File S3 summarises a detailed breakdown of the reporting quality for the 28 items of the CHEERS (2022) checklist. The median overall score was 20.5 [IQR = 18.4–21.6]. The reporting quality of the included studies was variable, ranging from 53.6–82.1% of the 28 items in the CHEERS checklists. The main reasons for the low quality of reporting included not fully reporting methods, results and other related information, not specifying the health economic analysis plan or rationale and a description of the model, not characterising heterogeneity and distributional effects and not engaging patients and others affected by the study.

### 3.3. Main Findings: Cost-Effectiveness of RRS

Overall, RRS is extremely cost-effective or cost-saving in most studies for women who are at a high risk of BC, intermediate/high risk of OC and high risk of EC, when compared with surveillance or no intervention. RRS remained cost-effective across a range of sensitivity analyses, and the results were robust and not affected much by the costs of surgery or treatment. However, the results were context-specific due to the diversity in terms of target populations, health systems and model assumptions and were sensitive to the disutility from RRS, age of RRS and uptake rates in terms of RRS. Additionally, RRESDO/OBS were sensitive to the uncertainty around effect sizes of OC risk reduction and long-term health impact.

#### 3.3.1. Cost-Effectiveness of RRM/RRSO in Unaffected *BRCA1/2* PV Carriers

For *BRCA1* PV carriers, two studies found that RRSO at the age of 35 was the most cost-saving [28] or cost-effective, with an ICUR of $2101/QALY [29], compared to other surgical prevention strategies (RRM or combined RRM and RRSO), chemoprevention (tamoxifen/oral contraceptives) and surveillance. Three other studies found that combined RRM and RRSO at the age of 40 [41], or combined RRM at the age of 35 with RRSO at the age of 45 [53] was the most cost-saving; RRM at the age of 30 with RRSO at the age of 35 was the most cost-effective, with an ICER of $835/LYG [47]. For *BRCA2* PV carriers, two studies found that combined RRM and RRSO at the age of 35 was the most cost-effective, with an ICUR of $3125/QALY [28], ant that combined RRM and RRSO at the age of 40 was the most cost-saving [41], compared with other preventive strategies or surveillance. A study reported that RRSO at the age of 40 was the most cost-effective, with an ICUR of $5535/QALY [29], and another one found that RRM at the age of 35 was the most cost-saving [53]. A familial cancer service program incorporating multidisciplinary clinic/RRM/RRSO/breast screening for unaffected *BRCA1/2* PV carriers was also found to be cost-effective compared to no intervention in one study using real world clinical data, with an ICUR of $23,353/QALY for *BRCA1* and $34,831/QALY for *BRCA2* [48]. The results were highly sensitive to the costs of RRM and uptake rates of RRSO, and the program was no longer cost-effective for *BRCA2* PV carriers if the uptake rates of RRSO were below 46%. For the chosen WTP threshold, this program was cost-effective in 98.2% of *BRCA1* simulations but only 40.6% of *BRCA2*.

For the combined analysis of *BRCA1* and *BRCA2* PV carriers, Grann et al. [44] found that RRM at the age of 30 was cost-effective, with an ICER ranging from $571/LYG to $2539/LY, and that RRSO or combined RRM and RRSO at the age of 30 was cost-saving, for the 40–85% BC-risk and 6–63% OC-risk used in their model. When utility scores for RRSO or combined RRM and RRSO were applied, only combined RRM and RRSO for the high-risk model (BC-85%, OC-63%) was cost-saving, while they did not report the data of related costs and QALYs. A key assumption of this study was that RRSO would reduce OC-risk by 50%. In the same study, the sensitivity analysis where the risk-reduction in terms of RRSO was assumed to be 90%, showed that the QALYs of RRSO and combined RRM and RRSO would increase. Moreover, if the utility score for RRSO increased from 0.91 to 0.98 or the utility score for combined RRM and RRSO increased from 0.86 to 0.93, the relevant strategy became cost-effective. On the other hand, Muller et al. [45] found that combined RRM and RRSO at the age of 30 was the optimal strategy compared to RRM and RRSO alone at the age of 30 or RRM at the age of 30 combined with RRSO at the age of 40. If the utility score for RRS increased to that of a healthy woman within a period of 25 years (5 years in the base-case), RRM at the age of 30 combined with RRSO at the age of 40 would be the dominant strategy.

The estimated QALY/LYG for RRS were quite heterogenous across the studies above, mainly due to the variation in the assumptions related to age of RRS, BC-risk reduction from RRSO, hormone replacement therapy (HRT) uptake after RRSO, disutility from RRS and separate/combined analysis for *BRCA1/2* PV carriers. Overall, absolute costs were found to be higher for *BRCA1* compared to *BRCA2* for all strategies. Most studies showed that a lower utility score for RRS and an increase in the duration of disutility related to RRS would make the RRS approach less cost-effective, but the ICER/QALY largely remained below the WTP cost-effectiveness thresholds.

#### 3.3.2. Cost-Effectiveness of RRM in Affected *BRCA1/2* PV Carriers with OC

For women affected by OC, RRM performed 5 years after OC diagnosis was only cost-effective for *BRCA1* PV carriers diagnosed with OC at the age of 40 years ($79,431/LYG), while this strategy was not cost-effective for *BRCA1* or *BRCA2* PV carriers diagnosed with OC at age ≥ 50 years, compared to breast screening [43]. RRM would become cost-effective after 5 and 8 years following OC diagnosis at the age of 50 in *BRCA1* or *BRCA2* PV carriers, respectively. The ICER of RRM performed five years later also increased (less cost-effective) with the older age of OC diagnosis and reduced BC-risk after OC treatment. PSA demonstrated that the probability for RRM performed 5 years later being cost-effective was only 38% and 13% in *BRCA1*- or *BRCA2*-associated OC patients diagnosed at the age of 50 years, respectively.

#### 3.3.3. Cost-Effectiveness of RRESDO in Unaffected *BRCA1/2* PV Carriers

Only one study evaluated the cost-effectiveness of RRESDO [36]. Although RRSO at the age of 40 was associated with the lowest cost and highest life expectancy, RRES at the age of 40 with DO at the age of 50 yielded the highest quality-adjusted life expectancy and was reported as being cost-effective, with an ICUR of $20,153/QALY and $27,498/QALY for *BRCA1* and *BRCA2* PV carriers compared to RRSO, respectively. RRES at 40 years alone had an ICUR of $18,118/QALY for *BRCA1* and $23,185/QALY for *BRCA2* PV carriers compared to RRSO, respectively. RRESDO would no longer be cost-effective when the utility score for RRSO exceeded 0.93 (from a base case of 0.82). However, a disutility for RRSO of 0.95 has also been reported in the literature [54]. Additionally, the base case assumed no HRT use after RRSO, which can bias results in favour of RRESDO. HRT use ranging from 40–75% has been reported in the literature [55,56]. The results were also sensitive to the age at which RRS was performed. Regarding the strategy where women underwent RRES at the age of 35 years with DO at the age of 46 years, this generated a favourable life expectancy compared to RRSO at the age of 40. The level of OC risk reduction from early-salpingectomy (ES) also influenced results, and, hence, the benefit of additional oophorectomy decreased if the level of risk reduction from ES increased, which finally resulted in an increase in the ICUR of RRESDO (less cost-effective). However, this may make RRES alone correspondingly more cost-effective.

#### 3.3.4. Cost-Effectiveness of Hysterectomy and BSO in Women with LS

In the three USA studies evaluating RRS in women with LS, OC- and EC risk were both considered in the model. However, only one of them incorporated colorectal cancer (CRC) risk [32]. Yang et al. [33] reported that combined hysterectomy and BSO at the age of 30 was cost-saving compared to annual gynecologic screenings or examination for women with LS, while they did not consider disutility of RRS. Their result was most sensitive to the cost of surgery and surgical mortality from hysterectomy and BSO, and the strategy remained cost-effective if it costs less than $106,826. Moreover, even with an older age in terms of hysterectomy and BSO, i.e., 70-years-old (compared to a base case of 30 years), the strategy remained cost-effective despite a diminished cost advantage over screening, and it was dominant in 99.97% of the simulations using PSA.

Kwon et al. [32] also found that combined hysterectomy and BSO at the age of 30 was cost-effective compared with no intervention, with an ICUR of $17,807/QALY, while hysterectomy and BSO at 40 years of age was found to be more cost-effective than that at the age of 30 years, with an ICUR of $6448/QALY. Despite the largest net health benefit being associated with the combined strategy of annual screening from 30 years with hysterectomy and BSO at 40 years, this was not cost-effective with an ICUR of $249,774/QALY compared to surgery alone [32]. The authors found that the highest health benefit was achieved when hysterectomy and BSO was conducted before 42 years of age in a combined strategy (screening and RRS). Furthermore, screening initiated at a later age after 30 would not make the combined strategy cost-effective, despite lower costs. The results were also highly sensitive to the utility score, as hysterectomy and BSO at 40 years of age would yield a higher health benefit than when performed at the age of 30 if its utility score was below 0.88.

Wright et al. [52] evaluated the gene-specific cost-effectiveness of RRS in women with LS. They found that hysterectomy and ES at the age of 40 with DO at the age of 50 was an optimal strategy for *MLH1* and *MSH6* PV carriers, with ICURs of $33,269/QALY and $20,008/QALY, respectively. Hysterectomy and BSO at the age of 40 was an optimal strategy for *MSH2* PV carriers, with an ICUR of $5180/QALY. Hysterectomy and BSO at the age of 50 was cost-saving for *PMS2* PV carriers. Importantly, compared with standard hysterectomy and BSO, two-stage ES and subsequent DO strategies in LS women came at the cost of an increased cancer incidence and mortality for the two-stage approach, which varied depending on the genetic variant. The increase in cancer incidence was *MLH1*: 7.76% vs. 3.84% and *MSH6*: 7.24% vs. 4.52%, and the increased cancer mortality was *MLH1*: 5.74% vs. 2.55% and *MSH6*: 5.22% vs. 2.97%. Hence, this is not ideal for maximizing cancer risk-reduction. The results were extremely sensitive to the disutility from early menopause and disutility from hysterectomy. They were also dependent on age at surgery and the effect-sizes in terms of OC risk-reduction from ES. There remains huge uncertainty in the literature regarding all these parameters. For *MLH1* and *MSH6* PV carriers, hysterectomy and ES at the age of 40 with DO at the age of 50 remained cost-effective, with utility scores for early menopause below 0.93 and 0.95, respectively. Otherwise, hysterectomy and BSO at the age of 40 or 35 would be the relevant optimal strategy. For *MSH2* PV carriers, the optimal strategy would be hysterectomy and ES at the age of 40 with DO at the age of 50, with utility scores for early menopause below 0.86, hysterectomy and BSO at the age of 40, with a utility score of 0.87–0.94, or hysterectomy and BSO at the age of 35, with a utility score greater than 0.95. If disutility of hysterectomy was taken into consideration, the optimal strategy would not change for *MSH2* or *MSH6* PV carriers, while hysterectomy and BSO at the age of 40 was favourable for *MLH1* PV carriers; no intervention was optimal for *PMS2* PV carriers, with a post-hysterectomy utility score less than 0.97 [52]. In PSA, the corresponding optimal strategy in the base case had a probability of 84.2% to be cost-effective for *MLH1*, 71.0% for *MSH6*, 86.2% for *MSH2* and 91.6% for *PMS2*.

#### 3.3.5. Cost-Effectiveness of RRSO in Women at Low/Intermediate OC Risk

Two UK-based studies evaluated the cost-effectiveness of RRSO separately for pre- and post-menopausal women across a range of OC risk thresholds at 2%, 4%, 5%, 6%, 8% and 10% [19,20]. For pre-menopausal women, RRSO at the age of 40 was cost-effective for a lifetime OC risk of 4% onwards, with an ICUR ranging from $32,164/QALY at 4% OC risk to $8283/QALY at 10% OC risk [19]. At the 4% risk-threshold, this corresponds to 10 life-years gained for a woman who may have developed OC. The base case results were highly sensitive to the utility score for RRSO, BC-risk reduction from RRSO and HRT compliance rate. Premenopausal RRSO would not be cost-effective, with the lowermost limit (95% confidence interval value) for an RRSO utility score for 4–7% OC risk thresholds, with the same being true without HRT after RRSO, at a ≤8% OC risk threshold, or without BC risk reduction from RRSO, at a <6% OC risk threshold. However, 37%, 61%, 74%, 84%, 96% and 99.5% simulations for PSA were cost-effective for pre-menopausal RRSO at the 2%, 4%, 5%, 6%, 8% and 10% levels in terms of OC risk, respectively.

For post-menopausal women, RRSO at the age of 51 was cost-effective for a lifetime OC risk threshold of 5% onwards, with an ICUR ranging from $25,103/QALY at 5% OC risk to $3069/QALY at 10% OC risk [20]. At the 5% risk threshold, this corresponds to seven life-years gained for a woman who may have developed OC. Regarding simulations for PSA, 67%, 80%, 84%, 91% and 94% of them were cost-effective for post-menopausal RRSO at risk thresholds of 4%, 5%, 6%, 8% and 10%, respectively. One-way sensitivity analysis suggested that these results were very sensitive to RRSO utility scores but not to treatment costs for RRSO or OC or heart disease.

The data illustrate the need for further research on utility scores associated with RRSO. The current estimates have a large standard deviation, and we need much better precision in terms of this estimate and an improved understanding of the quality of life impact. Additionally, separate utility scores are required for both pre- and post-menopausal women undergoing RRSO, given that the impact on quality of life may be different. This is not currently available, and utility scores are derived mainly from pre-menopausal women.

#### 3.3.6. Cost-Effectiveness of OBS in Women at Baseline Population OC Risk

Four studies evaluated the cost-effectiveness of OBS in women undergoing hysterectomy for benign gynaecologic conditions. OBS conducted along with hysterectomy (vaginal, laparoscopic or not specified) was found to be cost-effective, with an ICUR of $1667/QALY [46], or cost-saving [34,35,42] for OC prevention compared with hysterectomy alone. Switching from hysterectomy alone to hysterectomy with OBS could prevent one OC case in every 189/225/273 women and prevent one OC death in every 358/450 women [34,35,42]. Only one out of four studies incorporated a disutility of 0.77 from surgical complication over one year [34]. These results were sensitive to the increased-risk of subsequent benign adnexal surgery among women retaining tubes, while OBS with hysterectomy remained cost-effective even when excluding this variable [42]. The level of OC-risk reduction assumed in the base case in these studies varied from 50% to 65%, with most models (except one) using 65% from Falconer et al. [25]. Few studies undertook sensitivity analysis concerning the level of OC-risk reduction. Sensitivity analysis suggested that combined OBS with hysterectomy remains cost-effective if the level of OC risk reduction is higher than 31–33%. However, Falconer et al., in a more recent update of their earlier analysis [25], reported a much lower level of risk-reduction with salpingectomy, of 28%, after correcting for confounders such as pelvic inflammatory disease [26]. This highlights the need for prospective high-quality data to improve the precision of OC risk-reduction from OBS and establish its cost-effectiveness. OBS also appeared to be cost-effective as a result of sensitivity analysis if the complication rate rose up to 17.8%, if it costed up to $13,540 or the inadvertent oophorectomy risk during OBS was lower than 2% [34,35,46]. OBS was also found to be cost-saving compared with BSO with hysterectomy in pre-menopausal women, and if the age at hysterectomy exceeded 50.87 years, hysterectomy with BSO would become the dominant strategy [35]. Only Dilley et al. [34] conducted PSA, reporting a probability of 62.3% for OBS and hysterectomy to be cost-effective.

Six studies [34,35,46,49,50,51] included women who opted for permanent sterilisation; two [49,51] of them assessed permanent sterilisation in the context of a caesarean section. In the four (non-caesarean section) studies, only Dilley et al. [34] incorporated a disutility of 0.77 for surgical complication over one year. OBS was reported to be cost-saving [50] or cost-effective, with an ICER/ICUR ranging from $5469 to $33,883 per LYG or QALY [34,35,46], compared with other surgical sterilisation techniques including tubal ligation, clips or coagulation for reducing the risk of OC. Routine OBS at the time of permanent sterilisation could prevent one OC case in every 150/366/435 women, one OC death in every 834 women and one unintended pregnancy in every 1429 women compared with traditional surgical sterilisations [34,35,50]. Moreover, OBS was still cost-effective, with an OC risk reduction from OBS higher than 54% or at least a 25% increased reduction in OC risk compared to tubal sterilisation. Sensitivity analysis indicated that OBS would remain cost-effective for a complication rate of up to 2.5%, with cost below $7265 or less than that of tubal ligation by at least $1094, or with a pregnancy rate after OBS lower than 2.9% [34,35,46]. Specifically, one study found that OBS was no longer cost-effective compared to tubal coagulation if conducted at the age of 45 instead of 40 [50]. PSA results demonstrated that the probability for OBS being cost-effective ranged from 55–97% at the time of permanent sterilisation [34,50].

In the two studies evaluating the cost-effectiveness of OBS in the context of caesarean, they both found that OBS was cost-effective, with an ICUR of $28,109/QALY [49] and $24,490/QALY [51] compared to tubal ligation. OBS for permanent sterilisation during caesarean section could prevent one OC case in every 261/589 women, one OC-death in every 437/770 women and one unintended pregnancy in every 400/1134 women compared with tubal ligation [49,51]. The two models did not consider disutility from OBS or tubal ligation. Base case results were highly sensitive to OC risk reduction, procedure costs, complication rates and the failure rate in terms of OBS. OBS was no longer cost-effective if its cost was >$3341 more than that of tubal ligation or >$10,599, if the OC risk reduction regarding OBS was <41–52%, if the OC risk reduction regarding tubal ligation was >46%, if the complication rate regarding OBS was >9.9%, or if the failure rate regarding OBS was >50% [49,51]. PSA results demonstrated that OBS was cost-effective at the time of caesarean in roughly 50–70% of simulations [49,51]. The authors indicated that a preferred strategy could not be recommended before the level of OC risk reduction regarding OBS during caesarean section was well-defined.

## 4. Discussion

In this systematic review, we summarise the most updated evidence on the cost-effectiveness of RRS for the prevention of BC, OC and EC. Overall, RRS was extremely cost-effective or even cost-saving in certain scenarios compared to surveillance/no action in most studies for women with an increased risk of BC, OC and/or EC. For women with *BRCA1/2* PV, RRM and RRSO, both individually or in combination, were found to be cost-effective compared to surveillance/no action for unaffected women in eight studies, while RRM performed 5 years after OC diagnosis at age ≥ 50 years was not cost-effective compared to breast screening for affected women in one study [43]. The majority of these studies found combined RRM and RRSO was the most cost-effective strategy due to the highest life expectancy following RRS. The results were extremely sensitive to the disutility associated with RRS, the duration of this disutility, the age of surgery and the uptake of surgery. RRES at the age of 40 with DO at the age of 50 (RRESDO) was cost-effective compared to RRSO at the age of 40 in *BRCA1/2* PV carriers in only one study [36]. However, this model did not consider HRT use after RRSO, and hence may have underestimated the QALYs following RRSO. The results were extremely sensitive to the disutility associated with RRSO and the effect size in terms of OC risk reduction with RRES. There remains significant uncertainty around both these parameters. RRESDO is not cost-effective when the utility score for RRSO exceeds 0.93 (compared to the base case of 0.82). Importantly, a disutility of 0.95 for RRSO was reported in one study [54]. For women with LS, hysterectomy and BSO were cost-effective compared to surveillance in preventing EC and OC in three studies. Moreover, RRSO was found to be extremely cost-effective at the 4–5% lifetime OC risk level in a UK public healthcare setting. These interventions remained cost-effective across a range of different sensitivity analyses and were not significantly affected by the costs of surgery or treatment. Nevertheless, variables which significantly negatively impacted the overall cost-effectiveness included a higher disutility with RRSO, an older age in terms of RRS, lower uptake rates in terms of RRS, and lower levels of estimated OC risk reduction, particularly after salpingectomy.

For women at baseline population risk for OC, OBS combined with routine hysterectomy was found to be cost-effective or cost-saving compared to hysterectomy alone in four studies. These results were extremely sensitive to the level of OC risk reduction assumed with OBS. Most studies assumed a 65% reduction in OC risk. However, lower levels of OC risk reduction (less than 31–33%) and higher rates of complication from OBS may lead to a loss in cost-effectiveness. The results were also impacted by the age in terms of OBS and the route of hysterectomy, with vaginal hysterectomy being associated with higher complication rates. In terms of surgical sterilisation, OBS was reported to be cost-effective compared to other surgical techniques including tubal ligation/clips/coagulation in four studies. These results were also significantly influenced by the level of OC risk reduction, complication rates and the costs of OBS. Although OBS during caesarean section was cost-effective compared to tubal ligation in two USA studies, the uncertainty around OC risk reduction, complication rates and failure rates in terms of OBS in this specific context [57] made it difficult for strong recommendations to be made about the preferred strategy for sterilisation during caesarean section.

With respect to the methodology of economic evaluation, half of the studies performed both CEA and CUA, reporting LYG and QALY. Over a third of studies performed CUA only, and less than one fifth performed CEA only. Including utility scores would influence the cost-effectiveness of RRS, as disutility from these more invasive strategies would reduce the relevant QALYs generated. Over half of the studies developed a Markov model, with over one third developing a decision tree, and one with microsimulation. To evaluate the robustness of model outcomes, more than half of the studies performed both deterministic sensitivity analysis and PSA, with the remainder performing only a deterministic analysis. Parameters contributing to model uncertainty predominantly included the level of OC risk reduction from salpingectomy, the disutility of RRSO, the age in terms of RRS, RRS uptake rates and HRT compliance following premenopausal RRSO. The overall quality of the studies assessed using the CHEERS 2022 checklist ranged from 57.1–80.4% for RRM and/or RRSO, 64.3–82.1% for hysterectomy and BSO, 66.1% for RRESDO and 53.6–78.6% for OBS.

This is, to our knowledge, the largest and most updated systematic review on the cost-effectiveness of the surgical prevention of BC, OC and EC. This review included many more studies and surgical interventions than an earlier review which was restricted to North America and Europe and largely focused on the early detection and prevention of ovarian cancer [31]. Our review focused on risk-reducing surgeries for BC, OC and EC prevention. We identified important studies for RRM in OC-affected *BRCA1/2* PV carriers [43] and gene-specific analysis in women with LS [52], including studies from Japan [53] and Australia [48]. We used a robust methodology including a prospective protocol to report our findings according to PRISMA guidelines. We performed a detailed methodological appraisal of the included studies, highlighting limitations and knowledge gaps to help guide the design and delivery of future primary research and economic evaluation studies.

We used the most updated version of the CHEERS checklist, which provides a wider range of assessment in terms of reporting quality, giving a better insight into the interpretability of results. The median score was 20.5/28, indicating that the vast majority of the studies failed to report a significant amount of information, which is now considered important for the interpretation and implementation of study findings. In particular, few studies reported on distributional effects across different individuals within the population (e.g., variations in ethnicity, socio-economic status, etc.), few detailed whether their models were publicly available and no studies described approaches to engage stakeholders including patients or the public in the study design or following study findings. We do recognise that these criteria are being applied retrospectively, as all studies were published prior to the latest reporting guidelines in 2022. Nevertheless, future economic evaluations of preventive surgery should seek to address these where possible. In addition to the qualitative synthesis, to further enable a comparison across studies, the ICER/ICUR and cost threshold from sensitivity analysis reported in each study were inflated and converted to US$2020 using the CPI [38] of the original currency and PPP [39].

The current available evidence mainly applies to healthcare settings across high-income countries (North America, Europe, Japan and Australia). Analyses from middle- and lower-income country healthcare settings are lacking. Most analyses presented a payer perspective, half of them do not present a societal perspective or address productivity loss and a minority did not clarify the perspective. Due to the diversity in target populations, health systems and WTP thresholds across countries, the optimal RRS strategy and the age of surgery can vary and may be context specific. Due to significant heterogeneity in the methodology and reporting outcomes, we were unable to undertake a meta-analysis.

The findings regarding RRM and RRSO broadly support current practice recommendations regarding BC and OC preventive surgery for high-risk women in countries/regions including the UK [13,14,18], USA [58,59,60], Europe [61] and Australia [62]. Hysterectomy with BSO was found to be extremely cost-effective in women with LS, which is reflected in international recommendations [21]. Findings also support the use of OBS with gynaecological surgery and laparoscopic sterilisation. A discussion around OBS at hysterectomy is recommended by several societies, with a number of others not directly endorsing this [63]. Certain studies emphasise the lack of evidence around menopausal impact in pre-menopausal women and advocate for prospective trials prior to routine practice recommendations [64]. To our knowledge, there are no organisations recommending OBS at caesarean section, and whilst this was found to be cost-effective in two USA studies [49,51], the results were sensitive to the rates of OC risk reduction and peri-operative complications, which are uncertain.

There is a paucity of high-quality evidence for several critical parameters used in the cost-effectiveness models. In particular, the level of OC risk reduction with bilateral salpingectomy is not well defined. While data on OC risk reduction are available predominantly from retrospective population-based data sets following salpingectomy from Canada [65], the USA [66], Sweden [25] and Demark [27], these are predominantly retrospective, limited by a number of confounders and a small number of OC cases in the datasets and suffer from indication and detection biases [67,68]. Most cost-effectiveness models use the 65% reduction in OC risk from Falconer et al. in 2015 [25], but updated data from the same authors also indicate a 28% OC risk reduction after correcting for confounders of pelvic inflammation [26]. Prospective, well-designed data are needed to better define the precision of the estimated reduction in OC risk. The impact on long-term endocrine function is also unknown and needs addressing. The heterogeneity in the results and identified knowledge gaps stress the need for a prospective trial. Unfortunately, future RCTs will be difficult given the changes in clinical practice incorporating OBS and the resulting lack of equipoise. Most data will need to be derived from well-designed prospective cohort studies. Prospective clinical trials evaluating RRESDO for preventive surgery in women at increased OC risk are ongoing in the UK [23], Netherlands [22] and USA [24]. These will provide valuable evidence around quality of life, sexual function, menopause and costs to inform further economic evaluation. However, longer-term follow up will be needed to determine the level of OC risk reduction. Other important variables following salpingectomy during caesarean section are the rates in terms of peri-operative complications and completion of the surgery. This has been prospectively evaluated in small randomised trials [57,69] and retrospective studies [70]; however, larger studies are needed for greater precision in terms of these estimates and to inform future practice.

Disutility scores for RRM and RRSO are key parameters which can significantly affect cost-effectiveness modelling outcomes for RRS. However, the available evidence for utility scores following RRM and RRSO is limited. These are derived not from prospectively collected quality of life data from patients undergoing surgery but mainly from two studies involving vignettes valued by time trade-off exercises [54,71]. Such studies are subject to multiple biases and inconsistencies and great care is needed in their administration to avoid framing effects [72]. NICE recommends the development of utility scores from prospective evaluation of quality of life with standardised questionnaires such as EuroQol (EQ-5D) in women undergoing RRS. This approach is being addressed for RRSO/RRES in prospective trials [23].

The lifetime OC risk threshold at which RRSO is cost-effective has been evaluated and has led to a change in clinical practice [18,19,20], with more women now being able to access surgical prevention. This is particularly important given the lack of an effective screening strategy or national screening programme for OC [73]. However, the lifetime BC risk threshold for RRM has not yet been evaluated and established. Additional modelling and research are needed to define the BC lifetime risk threshold at which RRM should be offered. This is of critical importance, as personalised risk prediction models incorporating moderate penetrance genes, lifestyle factors, polygenic risk scores and mammographic density are increasingly being used for risk-adapted BC risk prediction and population stratification [74,75]. This can expand the use of RRM beyond *BRCA* PV carriers, which has been the focus of the majority of economic evaluations to date.

Among other cancer prevention strategies, screening (secondary prevention) is of paramount importance and should be compared with surgical prevention strategies. The shift in practice towards performing MRI for *BRCA1/2* PV carriers was considered in 6/8 of the studies included, while the cost-effectiveness of RRM varied when compared to surveillance. Three studies [41,45,53] reported that RRM was cost-effective or cost-saving compared to MRI-based screening. However, Grann et al. [29] found that mammography and MRI for *BRCA1/2* PV carriers was associated with highest costs, while its cost-effectiveness varied due to different disutility values when compared to RRM. RRM performed within 5 years of OC diagnosis at ≥50 years old was not found to be cost-effective compared to mammography and MRI screening for BC prevention [43]. More research is needed to address the difference in cost-effectiveness between RRM and mammography and MRI screening in *BRCA1/2* PV carriers for BC prevention using prospective disutility data for both interventions. As for OC and EC, there are no effective screening methods demonstrating mortality impact and, hence, no screening programmes available [76].

Recent therapeutic advances have led to PARPi being used for first line maintenance treatment of *BRCA1/2* mutated HER-2 negative BC and high-grade epithelial OC. Following initial improved disease-free survival in 2021 [77], increased overall survival was recently demonstrated on second interim analysis for HER-2 negative early BC (HR = 0.68; 98.5% CI = 0.47–0.97; *p* = 0.009) [78]. However, only progression-free survival has so far been demonstrated for OC (HR = 0.33; 95% CI = 0.25–0.43), with overall survival data remain unavailable [79,80]. The increased treatment costs and potentially improved survival of BC and OC care may influence the cost-effectiveness of surgical prevention. Only one of the included studies in our review considered the use of PARPi therapy in the treatment of ovarian cancer [48] and found preventive surgery to be cost-effective. The listed cost of Olaparib (PARP-i) is £2317.5/14-day pack in the UK and $13,886/30-day pack in the USA [81,82]. Given the high cost of PARPi treatment, it is likely that any analysis incorporating this into treatment costs will further improve the cost-effectiveness of preventive surgical interventions. Nevertheless, future studies should incorporate these innovative therapies for cancer treatment into relevant cost-utility analysis as mature data emerge. While this is possible now for BC [78], overall survival data for OC remain unavailable and need to be made available for robust cost-effectiveness analysis.

### Recommendations

1. High-quality prospective data is required for utility scores for patients undergoing RRS for BC and OC prevention as well as for preventive hysterectomy.
2. There is a need for large-scale prospective studies to generate high-quality evidence regarding the level of OC risk reduction and menopausal impact with respect to OBS and early-salpingectomy.
3. Further prospective evidence is required on the surgical morbidity and OC risk reduction in terms of OBS at caesarean section.
4. The lifetime BC risk threshold at which RRM is cost-effective needs to be established.
5. The costs and health effects of novel effective therapies such as PARPi should be incorporated into future cost-effectiveness modelling of surgical prevention strategies.
6. Economic evaluations of BC, OC and EC prevention should be undertaken in low- and middle-income countries and a broad range of health systems and contexts.
7. Given the recent update to the CHEERS checklist, the reporting quality of economic evaluations should meet the newly revised expectations. This will enable researchers to provide more uniformly reported data to facilitate further evidence synthesis and provide robust estimates from which to draw inferences.
8. There needs to be a move towards active and greater patient and public involvement in economic evaluation studies, including the dissemination of findings directly to patients as stakeholders and involving them actively in policy implementation.

## 5. Conclusions

This systematic review confirms that RRM and RRSO are cost-effective compared to surveillance/no action in women at a high risk of BC/OC (mainly for *BRCA1/2* PV carriers), with RRSO also being cost-effective in women at a 4–5% intermediate lifetime OC risk, and that changes in clinical guidelines need to reflect this. The novel strategy of RRESDO may potentially be cost-effective compared to RRSO, though the initial data are limited and the long-term outcome data needed for more robust modelling are forthcoming from ongoing trials. Moreover, hysterectomy and BSO is cost-effective in women with LS, supporting current international guideline recommendations. Although OBS appears to be a cost-effective option for OC risk reduction in the general population, this depends on the level of OC risk reduction. A key parameter impacting modelling results is the disutility from RRS. High-quality prospective data are needed to improve the precision of the estimate of the level of OC risk reduction following salpingectomy as well as the estimate in terms of disutility from RRSO, RRM and preventive hysterectomy.

## Figures and Tables

**Table 1:** Economic evaluation methodology.

Study	Economic Evaluation Type	Perspective	Study Design	Time Horizon	Sources for Costs	Sources for Effectiveness	Outcome Measures	Discount	Incremental Analysis	Sensitivity Analysis	CHEERS Checklist Score (28.0)
Anderson, 2006 [28]	CEA/CUA	Payer	Markov	Lifetime	Secondary/Literature	Primary data/Literature	LYG/QALY	Yes	Yes	One-way	21.0
Bommer, 2022 [41]	CEA/CUA	Payer	Markov	Lifetime	Secondary	Literature	LYG/QALY	Yes	Yes	One-way/PSA	22.0
Cadish, 2017 [42]	CEA	NR	Decision tree	NR	Secondary/Literature	Literature	Cancer/death prevented	No	No	One-way	17.0
Dilley, 2017 [34]	CUA	Payer	Decision tree	NR	Secondary/Literature	Literature	QALY	Yes	Yes	One-way/PSA	20.0
Gamble, 2017 [43]	CEA	Payer	Markov	Lifetime	Literature	Primary data/Literature	LYG	Yes	Yes	One-way/PSA	21.0
Grann, 1998 [44]	CEA/CUA	NR	Markov	50 years	Secondary	Primary data/Literature	LYG/QALY	Yes	Yes	One-way	16.0
Grann, 2011 [29]	CEA/CUA	Societal	Markov	Lifetime	Secondary/Literature	Primary data/Literature	LYG/QALY	Yes	Yes	One-way/PSA	19.0
Kwon, 2015 [35]	CEA	Societal	Markov	40 years	Secondary	Literature	LYG	Yes	Yes	One-way	19.5
Kwon, 2008 [32]	CUA	Societal	Markov	Lifetime	Secondary	Literature	QALY	Yes	Yes	One-way	18.0
Kwon, 2013 [36]	CEA/CUA	Societal	Markov	Lifetime	Secondary	Literature	LYG/QALY	Yes	Yes	One-way/two-way	18.5
Manchanda, 2016 [19]	CUA	Payer	Decision tree	Lifetime	Secondary/Literature	Literature	QALY	Yes	Yes	One-way/PSA	22.0
Manchanda, 2015 [20]	CUA	NR	Decision tree	Lifetime	Secondary	Literature	QALY	Yes	Yes	One-way/PSA	21.5
Muller, 2018 [45]	CEA/CUA	Payer	Markov	Lifetime	Primary data/Literature	Primary data/Literature	LYG/QALY	Yes	Yes	One-way/PSA	20.5
Naumann, 2021 [46]	CEA/CUA	Payer	Markov	Lifetime	Secondary/Literature	Literature	LYG/QALY	Yes	Yes	One-way	15.0
Norum, 2008 [47]	CEA	NR	Markov	Lifetime	Secondary/Literature	Literature	LYG	Yes	Yes	One-way	17.5
Petelin, 2020 [48]	CEA/CUA	Payer	Microsimulation	Lifetime	Secondary/Literature	Primary data/Literature	LYG/QALY	Yes	Yes	One-way/PSA	22.5
Subramaniam, 2019 [49]	CUA	Societal	Decision tree	Lifetime	Primary data/Literature	Primary data/Literature	QALY	Yes	Yes	One-way/two-way/PSA	20.5
Tai, 2018 [50]	CEA/CUA	Societal	Markov	Lifetime	Secondary/Literature	Literature	LYG/QALY	Yes	Yes	One-way/two-way/PSA	20.5
Venkatesh, 2019 [51]	CUA	Societal	Decision tree	Lifetime	Secondary/Literature	Literature	QALY	Yes	Yes	One-way/two-way/three-way/PSA	22.0
Wright, 2021 [52]	CEA/CUA	Payer	Markov	Lifetime	Secondary/Literature	Literature	LYG/QALY	Yes	Yes	One-way/PSA	23.0
Yamauchi, 2018 [53]	CEA/CUA	Societal	Markov	Lifetime	Primary data/Literature	Secondary/Literature	LYG/QALY	Yes	Yes	One-way	21.5
Yang, 2011 [33]	CUA	Societal	Decision tree	Lifetime	Secondary/Literature	Secondary/Literature	QALY	Yes	No	One-way/PSA	19.0

Sources of costs/effectiveness: primary data including questionnaires from individual patients; secondary data including unit cost lists. CEA, cost-effectiveness analysis; CUA, cost-utility analysis; LYG, life year gained; NR, not reported; PSA, probabilistic sensitivity analysis; QALY, quality-adjusted life year.

## Supplementary Materials

The following supporting information can be downloaded at: https://www.mdpi.com/article/10.3390/cancers14246117/s1, File S1: Search strategy; File S2: Study characteristics and main findings and important assumptions and limitations of included studies. File S3: CHEERS checklist score.
