# Supplementary material for: Cost-Effectiveness of Risk-Reducing Surgery for Breast and Ovarian Cancer Prevention: A Systematic Review
Source: Cancers (Basel). 2022 Dec 12;14(24):6117. doi: 10.3390/cancers14246117 (PMC9776851; doi:10.3390/cancers14246117)
Supplement: Supplementary file 1 [file cancers-14-06117-s001.zip › Supplementary File S1-v1.pdf]

Supplementary File S1. Search strategy.

1. Ovid MEDLINE

1 exp Prophylactic Surgical Procedures/
2 exp Mastectomy/
3 exp Ovariectomy/ or exp Salpingo-oophorectomy/
4 exp Salpingectomy/
5 ((prophylac* or prophylaxis or prevent* or risk-reduc* or risk reduc*) adj5 (surg* or procedur* or interven* or mastectom* or RRM or salping* or oophorectomy* or ovar* or RRSO or RRESDO)).mp.
6 1 or 2 or 3 or 4 or 5
7 exp Breast Neoplasms/
8 exp Ovarian Neoplasms/
9 exp Fallopian Tube Neoplasms/
10 exp Peritoneal Neoplasms/
11 ((ovar* or fallopian* or peritone* or breast or mammary) adj5 (cancer* or neoplasm* or tumor* or tumour* or malignan* or carcinoma* or adenocarcinoma*)).
12 7 or 8 or 9 or 10 or 11
13 exp Cost-Benefit Analysis/
14 (cost* or cost-effective* or cost-utilit* or cost-benefit* or CEA or CUA or CBA).mp.
15 13 or 14
16 6 and 12 and 15
17 limit 16 to (english language and humans)

2. Embase Classic+Embase

1 exp prophylactic surgical procedure/
2 exp prophylactic mastectomy/ or exp mastectomy/
3 exp salpingooophorectomy/
4 exp ovariectomy/
5 exp salpingectomy/
6 ((prophylac* or prophylaxis or prevent* or risk-reduc* or risk reduc*) adj5 (surg* or procedur* or interven* or mastectom* or RRM or salping* or oophorectomy* or ovar* or RRSO or RRESDO)).mp.
7 1 or 2 or 3 or 4 or 5 or 6
8 exp breast tumor/
9 exp ovary tumor/

10 exp uterine tube tumor/
11 exp peritoneum tumor/
12 ((ovar* or fallopian* or peritone* or breast or mammary) adj5 (cancer* or neoplasm* or tumor* or tumour* or malignan* or carcinoma* or adenocarcinoma*)).mp.
13 8 or 9 or 10 or 11 or 12
14 exp "cost effectiveness analysis"/
15 exp "cost utility analysis"/
16 exp "cost benefit analysis"/
17 (cost* or cost-effective* or cost-utilit* or cost-benefit* or CEA or CUA or CBA).mp.
18 14 or 15 or 16 or 17
19 7 and 13 and 18
20 limit 19 to (human and english language)

3. Cochrane Library
ID Search
#1 MeSH descriptor: [Mastectomy] explode all trees
#2 MeSH descriptor: [Salpingo-oophorectomy] explode all trees
#3 MeSH descriptor: [Ovariectomy] explode all trees
#4 MeSH descriptor: [Salpingectomy] explode all trees
#5 MeSH descriptor: [Prophylactic Surgical Procedures] explode all trees
#6 ((prophylac* or prophylaxis or prevent* or risk-reduc* or risk reduc*) near/5 (surg* or procedur* or interven* or mastectom* or RRM or salping* or oophorectomy* or ovar* or RRSO or RRESDO)):ti,ab,kw
#7 #1 or #2 or #3 or #4 or #5 or #6
#8 MeSH descriptor: [Breast Neoplasms] explode all trees
#9 MeSH descriptor: [Fallopian Tube Neoplasms] explode all trees
#10 MeSH descriptor: [Ovarian Neoplasms] explode all trees
#11 MeSH descriptor: [Peritoneal Neoplasms] explode all trees
#12 ((ovar* or fallopian* or peritone* or breast or mammary) near/5 (cancer* or neoplasm* or tumor* or tumour* or malignan* or carcinoma* or adenocarcinoma*)):ti,ab,kw
#13 #8 or #9 or #10 or #11 or #12
#14 MeSH descriptor: [Cost-Benefit Analysis] explode all trees
#15 (cost* or cost-effective* or cost-utilit* or cost-benefit* or CEA or CUA or CBA):ti,ab,kw

#16 #14 or #15
#17 #7 and #13 and #16

4. PubMed

1 prophylactic surgical procedure[MeSH Terms]
2 mastectomy[MeSH Terms]
3 salpingo-oophorectomy[MeSH Terms]
4 ovariectomy[MeSH Terms]
5 salpingectomy[MeSH Terms]
6 ((prophylac* or prophylaxis or prevent* or risk-reduc* or risk reduc*) near (surg* or procedur* or interven* or mastectom* or RRM or salping* or oophorectomy* or ovar* or RRSO or RRESDO))
7 breast neoplasm[MeSH Terms]
8 ovary neoplasm[MeSH Terms]
9 fallopian tube neoplasm[MeSH Terms]
10 peritoneal neoplasm[MeSH Terms]
11 (ovar* or fallopian* or peritone* or breast or mammary) near (cancer* or neoplasm* or tumor* or tumour* or malignan* or carcinoma* or adenocarcinoma*)
12 #1 or #2 or #3 or #4 or #5 or #6
13 #7 or #8 or #9 or #10 or #11
14 cost effectiveness[MeSH Terms]
15 cost benefit[MeSH Terms]
16 cost* or cost-effective* or cost-utilit* or cost-benefit* or CEA or CUA or CBA

17 #14 or #15 or #16
18 #12 and #13 and #17
