# Supplementary material for: Cost-Effectiveness of Risk-Reducing Surgery for Breast and Ovarian Cancer Prevention: A Systematic Review
Source: Cancers (Basel). 2022 Dec 12;14(24):6117. doi: 10.3390/cancers14246117 (PMC9776851; doi:10.3390/cancers14246117)
Supplement: Supplementary file 1 [file cancers-14-06117-s001.zip › Supplementary File S2-v1.pdf]

Table S1. Study characteristics and main findings.

Study	Country	Population	Risk profile	Lifetime cancer-risk	Age entering model	Intervention	Comparator	ICER/ICUR	Study reported conclusion
					**Women with *BRCA1/2* PV**				
Anderson, 2006 [1]	USA	Unaffected *BRCA1/2* PV-carriers	High-risk	*BRCA1*: BC-62%, OC-52% *BRCA2*: BC-76%, OC-23%	35	RRM at 35yo RRSO at 35yo RRM+RRSO at 35yo	Surveillance Chemoprevention	*BRCA1*: RRSO at 35yo was the most cost-saving *BRCA2*: RRM+RRSO at 35yo vs. RRSO at 35yo: $3,125/QALY	The most cost-effective strategies for *BRCA1* and *BRCA 2* PV-carriers were RRSO and combined RRSO and RRM, respectively.
Bommer, 2021 [2]	Switzerland	Unaffected *BRCA1/2* PV-carriers	High-risk	*BRCA1*: BC-72%, OC-44% *BRCA2*: BC-69%, OC-17%	40	RRM at 40yo RRSO at 40yo RRM+RRSO at 40yo	Surveillance Chemoprevention	RRM+RRSO at 40yo was the most cost-saving	Combined RRM and RRSO was the most cost-saving strategy for both *BRCA1* and *BRCA2* PV-carriers.
Grann, 1998 [3]	USA	Unaffected *BRCA1/2* PV-carriers from high-risk families	High-risk	High-risk model: BC-85%, OC-63% Intermediate-risk model, BC-56%; OC-16% Low-risk model: BC-40%, OC-6%	30	RRM at 30yo RRSO at 30yo RRM+RRSO at 30yo	Surveillance	LYG: 85% BC-risk/63% OC-risk: RRM at 30yo ($2,159/LYG), RRSO/RRM+RRSO at 30yo (cost-saving) 56% BC-risk/16% OC-risk: RRM at 30yo ($571/LYG), RRSO/RRM+RRSO at 30yo (cost-saving) 40%BC-risk/6% OC-risk: RRM at 30yo ($1,632/LYG), RRSO/RRM+RRSO at 30yo (cost-saving) QALY: only RRM+RRSO for high-risk model was cost-saving (data not reported)	RRM, RRSO, and combined RRM and RRSO was cost-effective using LYG compared with surveillance. RRSO and combined RRM and RRSO was not cost-effective using QALY compared with surveillance, except for the combined procedure in the high-risk model.
Grann, 2011 [4]	USA	Unaffected *BRCA1/2* PV-carriers	High-risk	Reported on annual BC/OC risk	30	RRM at 35yo RRSO at 35yo RRM+RRSO at 35yo RRSO+MRI at 35yo	Surveillance (mammography + MRI or mammography) Chemoprevention (tamoxifen)	*BRCA1*: RRSO at 35yo vs. RRM+RRSO at 35yo: $2,101/QALY *BRCA2*: RRSO at 35yo vs. RRM+RRSO at 35yo: $5,535/QALY	RRSO was the most cost-effective strategy for both *BRCA1* and *BRCA2* PV-carriers.
Kwon, 2013 [5]	Canada	Unaffected *BRCA1/2* PV-carriers	High-risk	*BRCA1*: BC-57%, OC-40% *BRCA2*: BC-49%, OC-18%	30	RRES at 40yo RRES at 40yo with DO at 50yo	RRSO at 40yo	*BRCA1*: RRES at 40yo with DO at 50yo vs. RRSO at 40yo: $20,153/QALY RRES at 40 yo vs. RRSO at 40yo: $18,118/QALY *BRCA2*: RRES at 40yo with DO at 50yo vs. RRSO at 40yo: $27,498/QALY RRES at 40 yo vs. RRSO at 40yo: $23,185/QALY	RRESDO was a potentially cost-effective strategy and may be an acceptable alternative for those unwilling to undergo RRSO.

Muller, 2018 [6]	Germany	Unaffected *BRCA1/2* PV-carriers	High-risk	Reported on annual BC/OC risk	30	RRM at 30yo<br>RRSO at 30yo<br>RRM+RRSO at 30yo<br>RRM at 30yo + RRSO at 40yo	Surveillance	RRM +RRSO at 30yo was the most cost-saving	Combined RRM and RRSO was the most cost-effective strategy for BRCA1/2 PV-carriers.
Norum, 2008 [7]	Norway	Unaffected *BRCA1* PV-carriers	High-risk	BC-58%, OC-58%	30	RRM at 30yo + RRSO at 35yo<br>RRSO at 35yo	Conservative (no action)	RRM at 30yo + RRSO at 35yo vs. no action: $835/LYG<br>RRSO at 35yo vs. no action: $2,162/LYG	RRSO with or without RRM in *BRCA1* PV-carriers was cost-effective.
Petelin, 2020 [8]	Australia	Unaffected *BRCA1/2* PV-carriers	High-risk	Reported on annual BC/OC risk	20	Combined surveillance & risk-reduction Program (multidisciplinary clinic/RRSO/RRM/ breast screening)	Conservative (no action)	*BRCA1*: $23,353/QALY<br>*BRCA2*: $34,831/QALY	Long-term management of *BRCA1/2* PV-carriers within a structured risk management & surveillance program was cost-effective.
Yamauchi, 2018 [9]	Japan	Unaffected *BRCA1/2* PV-carriers	High-risk	Reported on annual BC/OC risk	35	RRM at 35yo<br>RRSO at 45yo<br>RRM at 35yo + RRSO at 45yo	Surveillance	*BRCA1*:<br>RRM at 35yo +RRSO at 45yo was the most cost-saving<br>*BRCA2*:<br>RRM at 35yo was the most cost-saving	Combined RRM and RRSO was the most cost-effective strategy in *BRCA1* PV-carriers, and RRM was the most cost-effective strategy in *BRCA2* PV-carriers.
Gamble, 2017 [10]	USA	Affected *BRCA1/2* PV-carriers with OC	High-risk	Not reported	40	RRM (5-years after OC diagnosis)	Surveillance (MRI + mammography)	*BRCA1:*<br>OC diagnosed at 40yo: RRM vs. surveillance: $79,431/LYG<br>OC diagnosed at 50yo: RRM vs. surveillance: $119,700/LYG<br>*BRCA2:*<br>OC diagnosed at 40yo: RRM vs. surveillance: $113,090/LYG<br>OC diagnosed at 50yo: RRM vs. surveillance: $183,982/LYG	RRM performed within 5-years following OC diagnosis at ≥50yo was not cost-effective. Surveillance should be advocated during the first several years after OC diagnosis, after which point the benefits of RRM can be considered based on patient age and *BRCA* status.
					**Women with LS**				
Kwon, 2008 [11]	USA	Women with LS	High-risk	EC-50%, OC-10%	30	Hysterectomy + BSO at 30/40yo<br>Annual screening from 30yo until hysterectomy + BSO at 40yo<br>Annual screening from 30yo	Conservative (no action)	Hysterectomy + BSO at 30yo vs. no action: $17,807/QALY<br>Hysterectomy + BSO at 40yo vs. Hysterectomy + BSO at 30yo: $6,448/QALY<br>Combined strategy vs. Hysterectomy + BSO at 40yo: $249,774/QALY<br>Annual screening vs. Combined strategy: dominated	Annual screening followed by hysterectomy and BSO at 40yo was the most effective strategy, but the incremental benefit over prophylactic surgery alone was attained at substantial cost.
Wright, 2021[12]	USA	Women with LS (*MLH1/MSH*	High-risk	EC-48.9%<br>OC-17.4%	25	Hysterectomy + BSO at 35/40/50yo	Conservative (no action)	*MLH1/MSH6*:<br>Hysterectomy + ES at 40yo with DO at 50yo	For *MSH2* PV-carriers, hysterectomy and

		2/*MSH6*/*PMS2* PV-carriers)				with or without surveillance from 35yo Hysterectomy + ES at 40yo with DO at 50yo		vs. Hysterectomy + BSO at 40yo: $33,269/20,008/QALY *MSH2*: Hysterectomy + BSO at 40yo vs. Hysterectomy + BSO at 35yo: $5,180/QALY *PMS2*: Hysterectomy + BSO at 50yo vs. Hysterectomy + BSO at 40yo: cost-saving	BSO at 40yo was the most cost-effective strategy; for *MLH1* and *MSH6* PV-carriers, hysterectomy and ES at 40yo with DO at 50yo was the most cost-effective strategy; for *PMS2* PV-carriers, hysterectomy and BSO at 50yo was the most cost-effective strategy.
Yang, 2011 [13]	USA	Women with LS	High-risk	EC: 40-60% OC: 10%-12%	30	Hysterectomy + BSO at 30yo	Surveillance (gynaecologic screening/examination)	Hysterectomy + BSO at 30yo was cost-saving	Hysterectomy and BSO was the most cost-effective strategy in women with LS.
				**Women at low/intermediate OC-risk**					
Manchanda, 2016 [14]	UK	Premenopausal women	Low/intermediate-risk	OC-2%, 4%, 5%, 6%, 8%, 10%	40	RRSO at 40yo	Conservative (no action)	4% OC-risk: $32,164/QALY 10% OC-risk: $8,283/QALY	Premenopausal RRSO was cost-effective at ≥4% lifetime OC-risk.
Manchanda, 2015 [15]	UK	Postmenopausal women	Low/intermediate-risk	OC-2%, 4%, 5%, 6%, 8%, 10%	51	RRSO at 51yo	Conservative (no action)	5% OC-risk: $25,103/QALY 10% OC-risk: $3069/QALY	Postmenopausal RRSO was cost-effective at≥5% lifetime OC-risk.
				**Women at baseline population OC-risk**					
Cadish, 2017 [16]	USA	Women having vaginal hysterectomy	Baseline-risk	OC-1.3%	Not reported	Vaginal hysterectomy + OBS	Vaginal hysterectomy	Vaginal hysterectomy + OBS was cost-saving	OBS was cost-effective strategy and should routinely be performed with vaginal hysterectomy.
Dilley, 2017 [17]	USA	Women having laparoscopic hysterectomy or sterilization	Baseline-risk	OC-1.3%	45 (Laparoscopic Hysterectomy) 35 (Laparoscopic sterilization)	Model 1: Laparoscopic hysterectomy + OBS Model 2: Laparoscopic OBS	Model 1: Laparoscopic hysterectomy Model 2: Laparoscopic sterilization	Laparoscopic hysterectomy + OBS vs. Hysterectomy: cost-saving Laparoscopic OBS vs. Tubal occlusion: $33,883/QALY	OBS for women undergoing hysterectomy or opting for permanent contraception may be a cost-effective strategy for reducing OC-risk. OBS was cost-effective with both procedures, but the advantage greater at time of hysterectomy.
Kwon, 2015 [18]	Canada	Premenopausal women having hysterectomy or surgical sterilization	Baseline-risk	OC-1.4%	45 (Hysterectomy) 35 (Surgical sterilization)	Model 1: Hysterectomy + OBS Hysterectomy + BSO Model 2: OBS	Model 1: Hysterectomy Model 2: Tubal ligation	Hysterectomy + OBS vs Hysterectomy: cost-saving OBS vs. Tubal ligation: $23,951/LYG	Hysterectomy with OBS for benign conditions would reduce OC-risk at acceptable cost and was a cost-effective alternative to tubal ligation for sterilization.
Naumann, 2021 [19]	USA	Women having laparoscopic	Baseline-risk	Reported on annual OC-risk	20	Model 1:	Model 1: Laparoscopic hysterectomy	Laparoscopic hysterectomy + OBS vs.	Both strategies are cost-effective when

		hysterectom y or tubal ligation				Laparoscopic hysterectomy + OBS Model 2: OBS	Model 2: Tubal ligation	Hysterectomy: $1,667/QALY OBS vs. Tubal ligation: $5,469/QALY	considering the cost of OBS alone.
Subramani am, 2019 [20]	USA	Women opting for permanent sterilization at the time of caesarean delivery	Baseline-risk	OC-1.26%	33	OBS	Tubal ligation	OBS vs. Tubal ligation: $28,109/QALY	In women undergoing caesarean delivery with sterilization, OBS was likely cost-effective and may be cost-saving in comparison to tubal ligation for OC-risk reduction.
Tai, 2018 [21]	Canada	Women opting for permanent sterilization	Baseline-risk	OC-1.5%	40	Laparoscopic OBS	Laparoscopic tubal coagulation/ tubal clips	Laparoscopic OBS was cost-saving	Laparoscopic OBS was the most cost-effective strategy when compared to tubal clips/tubal coagulation to prevent OC.
Venkatesh, 2019 [22]	USA	Women opting for permanent sterilization at the time of caesarean delivery	Baseline-risk	OC-1.28%	35	OBS	Tubal ligation Long-acting reversible contraception	OBS vs. Tubal ligation: $24,490/QALY	OBS with caesarean delivery were cost-effective for permanent sterilization and OC-risk reduction, but the risks/benefits of salpingectomy with caesarean delivery need to be better defined before a preferred strategy can be determined.

BC, breast cancer; BSO, bilateral salpingo-oophorectomy; DO, delayed oophorectomy; EC, endometrial cancer; ES, early-salpingectomy; ICER, incremental cost-effectiveness ratio; ICUR, incremental cost-utility ratio; LS, Lynch syndrome; LYG, life-years gained; MRI, magnetic resonance imaging; OBS, opportunistic bilateral salpingectomy; OC, ovarian cancer; PV, pathogenic variant; QALY, quality-adjusted life-years; RRES, risk-reducing early salpingectomy; RRESDO, risk-reducing early salpingectomy with delayed oophorectomy; RRM, risk-reducing mastectomy; RRSO, risk-reducing salpingo-oophorectomy; yo, years old.

Table S2. Important assumptions and limitations of included studies.

Studies	Additional study reported assumptions	Study reported limitations*	Additional points on Critical Review#
		**Women with *BRCA1/2* PV**	
Anderson, 2006 [1]	• Annual conditional cancer risks were converted from 10-year risks by assuming constant instantaneous rates of disease per-year within each decade. • The proportion of patients who currently had metastatic BC or OC was assumed to be equal to the proportion of patients expected to die in the subsequent 3 years. • The conditional probability of death in *BRCA1/2*-associated cancer was the same as that in cancer in general population. • The BC risk-reduction of RRM was 90% without RRSO and 95% with RRSO; the BC risk-reduction of RRSO was 45%; the OC risk-reduction of RRSO was 96%. • All premenopausal women would have HRT after RRSO, and it would not increase risk of BC, cardiovascular disease and osteoporosis. • Costs of genetic testing and counselling were included. • Utility of RRM was 0.76; RRSO was 0.82; both surgeries was 0.76 [23].	• The accuracy of model assumptions was uncertain. • The risks of HRT for pre-menopausal women were unknown.	• Separate analysis for *BRCA1/BRCA2* was performed. • Reconstruction after RRM was not considered. • Compliance with HRT was not considered. • BC risk-reduction from RRSO was modelled at 45%. • Impact of premenopausal RRSO on heart disease was not modelled. • One-way sensitivity analysis was undertaken but PSA not done.
Bommer, 2022 [2]	• The distribution of the BC molecular subtypes HR+, TNBC and Her2+ were 20%, 70%, 10% for *BRCA1* and 80%, 10%, 10% for *BRCA2*. • 4.38% of women diagnosed with BC had a nodal involvement that required radiotherapy. • 6.63% of women had residual disease after OC surgery and 73% of women relapsed within 2-years. • Uptake rate of RRS and HRT after RRSO was 100%. • 5% and 95% of women after RRM chose autologous and implant-based breast reconstruction, respectively. • The BC risk-reduction of RRM was 91%; the OC risk-reduction of RRSO was 72%. RRSO did not reduce BC risk. • A disease-related lower utility score than in disease-free state was assigned for the first 5-years after cancer diagnosis, after which they returned to an age-adjusted disease-free utility unless patients were ill again with cancer or died. • Utility of RRM was 0.88; RRSO was 0.90; both surgeries was 0.79 [24]. The disutility was included for one-year.	• Age-specific BC death rates of Switzerland were used regardless of the molecular subtype or *BRCA*-status as corresponding Swiss data was missing. • Costs may be underestimated as costs of diagnostic biopsies, side effects and complications of chemoprevention, and costs of consultation were not included. • Compliance with HRT was not considered.	• Separate analysis for *BRCA1/BRCA2* was performed. • Deterministic and probabilistic sensitivity analysis was undertaken. • OC risk-reduction from RRSO was only 72%. • Disutility for RRM and RRSO was included for only one-year. • Impact of premenopausal RRSO on heart disease was not modelled. • Annual gynaecological USS was included in surveillance (but this has no clinical benefit [25]).
Grann, 1998 [3]	• 20% of women diagnosed with BC was node-positive. • Annual conditional probabilities of cancer were converted from 10-year risks by assuming constant instantaneous rates of disease per-year within each decade. • The proportion of patients who currently had metastatic BC or OC was assumed to be equal to the proportion of patients expected to die in the subsequent 3 years. • Cancer patients 10 years post-diagnosis had the same conditional probability of death as the general population. • The BC risk-reduction of RRM was 90%; the OC risk-reduction of RRSO was 50% in the base-case. • Women had reconstruction after RRM. • All premenopausal women would have HRT after RRSO, and it would not increase risk of BC, cardiovascular disease and osteoporosis. • Utility of RRSO was 0.91; both surgeries was 0.86 (from a time-trade-off survey by authors). Disutility of RRM was not included as they assumed most women chose RRSO or both surgeries.	• The utility scores measured in community-based population survey, may be unduly low and did not take into account the reduction in anxiety that patients with FH derive from RRS.	• No separate analysis was performed for *BRCA1/BRCA2*. • Compliance with HRT was not considered. • Impact of premenopausal RRSO on heart disease was not modelled • OC risk-reduction of RRSO was only 50%. • *BRCA1* risks for BC and OC were too high at 85% and 63% respectively (though reflected data at the time). • One way sensitivity analysis was performed but PSA not undertaken.
Grann, 2011 [4]	• Annual conditional probabilities of cancer were converted from 10-year risks by assuming constant instantaneous rates of disease per-year within each decade.	• There was lack of data regarding the assumptions used about	• Separate analysis for *BRCA1/BRCA2* was performed.

	• *BRCA1/2* PV-carriers with BC had the same conditional probability of developing OC as those who were well.<br>• The conditional probability of death in *BRCA1/2*-associated cancer was the same as that in cancer in general population.<br>• The stage distribution at BC diagnosis for women who received MRI was the same as that of the cases in a cohort who received annual MRI and mammography. The stage distribution among women who had mammography alone would be the same as the one for high-risk women in the control arm of a tamoxifen trial.<br>• The BC risk-reduction of RRM was 90% without RRSO and 95% with RRSO; the BC risk-reduction of RRSO was 47%; the OC risk-reduction of RRSO was 96%.<br>• HRT after RRSO does not increase the risk of BC, cardiovascular disease and osteoporosis.<br>• Utility of RRM was 0.88; RRSO was 0.90; both surgeries was 0.79 [24].	stage distribution at BC diagnosis, for women under MRI and mammography surveillance versus mammography alone.	• Reconstruction after RRM was not considered.<br>• Compliance with HRT was not considered.<br>• Impact of premenopausal RRSO on heart disease was not modelled.<br>• BC risk-reduction from RRSO was modelled at 47%.<br>• One way sensitivity analysis was undertaken but PSA not provided.
Kwon, 2013 [5]	• 50% of women diagnosed with BC would choose bilateral mastectomy, and the other half would choose breast-conserving surgery.<br>• All women diagnosed with OC would undergo laparotomy, hysterectomy, BSO, and debulking or other staging surgery.<br>• Cancer patients 10 years post-diagnosis would have the same conditional probability of death as the general population.<br>• 30% of *BRCA1/2* PV-carriers would choose to undergo RRM and following reconstruction, ranging from 21% between the ages of 25 and 60 years to 34% between the ages of 23 and 64 years.<br>• Uptake rate of RRS was 100%, and women did not have HRT after RRSO.<br>• The BC risk-reduction of RRSO was 40% for *BRCA1* and 70% for *BRCA2*; there was no BC risk-reduction from salpingectomy. The OC risk-reduction of RRSO was 80%; the OC risk-reduction of salpingectomy was 60%.<br>• Utility of RRM was 0.86; RRSO was 0.82; both surgeries was 0.79 [23]. Utility of salpingectomy was set at 0.99.	• The QALY of RRSO may be underestimated as no HRT was used after RRSO.<br>• There were limited data on proportion of *BRCA*-associated OC that primarily arise in fallopian tube and benefits/risks of salpingectomy.<br>• There was uncertainty related to the disutility of RRS and healthcare costs.	• Separate analysis for *BRCA1/BRCA2* was performed.<br>• Disutility of salpingectomy was set at 0.01. This is an assumption as these data are not available.<br>• BC risk-reduction from RRSO was modelled at 40-70% and may be overestimated.<br>• The true effect size and precision of OC risk-reduction from early salpingectomy is unknown and can affect overall results.<br>• Results are highly sensitive to the disutility of RRSO. The disutility of RRSO in the base case appears high at 0.82. A disutility of RRSO of 0.95 [24] has also subsequently been reported in the literature by the same authors reporting 0.82 initially [23].<br>• RRESDO may not be cost-effective when the utility score of RRSO exceeds 0.93.<br>• The base-case assumed no HRT use after RRSO, which can bias results in favour of RRESDO. HRT use ranging from 40-75% has been reported in the literature [26,27].
Muller, 2018 [6]	• Women with BC who did not die or develop a recurrent/contralateral/metastatic BC or develop OC moved to a post-BC state with gradually increasing utility; follow-up and treatment costs were calculated up to year 5 post BC diagnosis. From the sixth year onwards, women stayed in the post-BC state unless another cancer event occurred, with no treatment costs.<br>• Women with recurrent BC or local/regional spread returned to the initial BC state with treatment costs assumed to be the same as for their first BC.<br>• A state for recurrent/metastatic OC was not included due to a lack of stage-specific data and the expected high mortality rate of OC.<br>• Cancer-specific death was assumed to occur only in metastatic BC and OC.<br>• The BC risk-reduction of RRM was 92% without RRSO and 95% with RRSO; the BC risk-reduction of RRSO was 47%; the OC risk-reduction of RRSO was 72%.	• Data accuracy was limited.<br>• Although BC and OC risks differ by *BRCA* subtype, this model combined *BRCA 1/2* PV-carriers.<br>• The QALY of RRSO may be underestimated as no HRT was used after RRSO.	• No separate analysis was performed for *BRCA1/BRCA2*.<br>• Costs may be underestimated due to the assumption that the costs of recurrent/spread BC were the same as the first BC.<br>• Reconstruction after RRM was not considered.<br>• BC risk-reduction from RRSO was modelled at 47%.<br>• OC risk-reduction from RRSO is underestimated.<br>• Utility of RRS may be a bit lower than estimated.<br>• HRT after RRSO was not considered.

	• Women did not have HRT after RRSO. • Utility declined as a result of BC or OC and then increased linearly for 5 years to reach the age-specific utility of a post-cancer state, while for metastatic BC and end-stage OC, a permanent decrease in utility was assumed. • Utility of RRM was 0.85; RRSO was 0.83; both surgeries was 0.78 [23] . Disutility of RRS was assumed to increase in a linear manner for 5 years to regain the age-specific utility of an otherwise healthy woman with *BRCA1/2* PV.		• Impact of premenopausal RRSO on heart disease was not modelled • Both one-way and PSA was undertaken.
Norum, 2008 [7]	• The prognosis of *BRCA1*-associated BC in Norway was poor with almost no benefit of early diagnosis or adjuvant chemo/radiotherapy. • The BC risk-reduction of RRM was ~85% (down to baseline-risk); the BC risk-reduction of RRSO was 50%; the OC risk-reduction from RRSO was 90%. • All premenopausal women had HRT after RRSO. • Half of the *BRCA1* PV-carriers were in their routine workplace for seven more years. • Productivity loss was included in the analysis.	• Acceptance of RRM was unknown. • Some assumptions may be debatable, including prognosis of *BRCA*-associated BC.	• Only *BRCA1* analysis was performed, with *BRCA2* analysis missing. • Disutility was missing from the model. This is a significant omission. • The outcome measure was life-years gained; utility was not included. • Reconstruction after RRM was not considered. • BC risk-reduction from RRSO was modelled at 50% and is likely overestimated. • Compliance with HRT was not considered. • Impact of premenopausal RRSO on heart disease was not modelled. • PSA was not undertaken though one-way sensitivity analysis provided.
Petelin, 2020 [8]	• Cancer recurrence/remission were not modeled. • Cancer-affected women were at risk of cancer-specific death for 10 years from diagnosis. • *BRCA*-associated BC mortality were the same as the general BC population. • PARP-I was included only for relapsed OC. • Cancer-affected women attend annual specialist follow-up for 10 years from their diagnosis. • Women had reconstruction after RRM. • The BC risk-reduction of RRM was 93%; the BC risk-reduction of RRSO was 0 for *BRCA1* and 83% for *BRCA2*; the OC risk-reduction of RRSO was 81%. • RRSO increased risk of mortality due to cardiovascular disease by 3%. • Utility of RRM was 1. Utility for RRSO was 0.95 [24].	• There was uncertainty around cancer treatment costs, utility score, and the unobservable aspects of *BRCA*-specific natural history for BC and OC. • It was unlikely that women with *BRCA* PV would not take any action, so the choice of comparator of "no-action" may overestimate the cost-effectiveness of the service program.	• Separate analysis for *BRCA1*/*BRCA2* was performed. • Use of HRT after RRSO was not modelled. • Impact of premenopausal RRSO on heart disease was not modelled • RRM was assumed to cause no disutility. • Disutility with RRSO was short-term only. • One way sensitivity analysis and PSA were undertaken.
Yamauchi, 2018 [9]	• Women had reconstruction after RRM. • The BC risk-reduction of RRM was 90%; the OC risk-reduction of RRSO was 96%. RRSO did not reduce BC-risk. • Utility of RRM was 0.88; RRSO was 0.90; both surgeries was 0.79 [24] .	• The BC and OC morbidity among *BRCA1/2* PV-carriers, BC recurrence rates, cancer risk-reduction from RRS, and utility scores were derived from studies conducted outside Japan.	• Separate analysis for *BRCA1*/*BRCA2* was performed. • Use of HRT after RRSO was not modelled. • Impact of premenopausal RRSO on heart disease was not modelled • PSA was not undertaken but one-way sensitivity analyses provided.
Gamble, 2017 [10]	• Women who remained alive following OC diagnosis remained at risk of developing BC until cancer-related death (OC) or death from other causes. • Women were considered long-term OC survivors if (1) they had no disease recurrence or progression 7 years after OC diagnosis, or (2) they were alive 15 years after OC recurrence. Long-term OC survivors were considered to no longer be at risk of death from OC.	• Previously published data from different years in different populations was used. • Other preventive strategies including chemoprevention and	• An important issue of RRM in *BRCA* women with OC was addressed. • Separate analysis for *BRCA1* and *BRCA2* was performed. • The outcome measure was life-years gained; utility was not included.

	• Women were considered long-term BC survivors if they were alive without evidence of recurrence 15 years after BC diagnosis. Long-term BC survivors were considered to no longer be at risk of death from BC.<br>• Among *BRCA* PV-carriers, 80% would be TNBC and 20% would be non-TNBC.<br>• The conditional probability of death in *BRCA*-associated cancer was the same as that in cancer in general population.<br>• RRM was performed 1-15 years after OC diagnosis, assuming no disease recurrence/progression.<br>• 82% of women undergoing RRM would undergo either immediate/delayed reconstruction.<br>• The BC mortality was reduced by 71% from RRM.	disutility of RRM were not included.	• One way sensitivity analysis and PSA were undertaken.
	**Women with LS**		
Kwon, 2008 [11]	• All women were at risk for both EC and OC, and these risks were "eliminated" (down to baseline risk) after hysterectomy and BSO.<br>• If EC or OC was missed during screening or diagnostic testing, then the diagnosis was made within the next year.<br>• The lifetime risk of CRC was also included.<br>• Compliance with interventions was 100%.<br>• Utility of BSO was 0.86 [23].	• Empiric data on screening and utility scores was lacking.	• Use of HRT after BSO was not modelled.<br>• Impact of premenopausal RRSO on heart disease was not modelled<br>• The true disutility with preventive hysterectomy is unknown.<br>• The disutility of 0.86 modelled was only till age 45 years.<br>• PSA was not undertaken though one way sensitivity analyses were presented.
Wright, 2021 [12]	• EC and OC diagnoses were independent to each other, and when one of the two cancer diagnoses was made, a patient was no longer at risk of developing the other cancer.<br>• EC and OC risk was "eliminated" (down to baseline risk) after hysterectomy and BSO. EC risk after hysterectomy and ES at age 40 years was eliminated with no OC-risk reduction, so patients remained at risk for OC until DO was performed at age 50 years.<br>• Compliance with interventions was 100%.<br>• Anyone receiving a false-positive result from OC screening would immediately undergo hysterectomy and BSO.<br>• No added risk of all-cause mortality associated with early menopause after RRSO was included.<br>• Disutility of early menopause was 0.1 till age 50 years; disutility of complication was 0.0425 and of convalescence was 0.025 from RRS for one year.	• Lack of data on individual or FH of LS-associated cancers, limited the use of microsimulation.<br>• Other LS-associated cancers were not included, given primary focus at gynecologic cancer.	• This is important analysis providing gene specific cost-effectiveness data in women with LS.<br>• Possible OC over-diagnosis during screening.<br>• Use of HRT after BSO was not modelled.<br>• Impact of premenopausal RRSO on heart disease was not modelled.<br>• The demonstration of cost-effectiveness of two-stage strategy of ES and DO in LS women, compared with standard hysterectomy and BSO comes at the cost of an increased cancer incidence and mortality for the two-stage approach which varied dependent on the genetic variant. Hence, this is not ideal for maximizing cancer risk-reduction.<br>• There remains significant uncertainty on the effect size of cancer risk-reduction with ES and the disutility with surgical risk-reduction, both of which can significantly impact outcomes.<br>• Both one way sensitivity analysis and PSA were presented.
Yang, 2011 [13]	• A decision on colorectal surveillance and prevention would be separate and independent from the discussion of gynecologic cancer risks. CRC were not modelled.<br>• Women with an abnormal endometrial biopsy including hyperplasia or carcinoma would undergo hysterectomy and BSO.<br>• Women with either persistently abnormal findings on pelvic ultrasound, or a combination of elevated CA125 and abnormal pelvic ultrasound would undergo further diagnostic tests and ultimately have hysterectomy and BSO, or return to annual surveillance.	• Models cannot represent every detail of clinical practice.<br>• Some data was not based on women with LS due to limited patient numbers.<br>• The impact of non-gynecologic LS-associated malignancies on	• Disutility for hysterectomy and BSO was not incorporated.<br>• Use of HRT after BSO was not modelled.<br>• Impact of premenopausal RRSO on heart disease was not modelled.<br>• PSA was not undertaken.

		mortality was not included.	
**Women at low/intermediate OC-risk**			
Manchanda, 2016 [14]	• Compliance with HRT was 80%, and short-term HRT did not affect BC-risk.<br>• The chance of both BC and OC occurring at the same time was rare and assumed to be close to zero.<br>• Cancer patients 10 years post diagnosis had the same conditional probability of death as the general population.<br>• Costs for identifying/calculating OC-risk were not included.<br>• The BC risk-reduction of RRSO was 38%; the OC risk-reduction of RRSO was 94%.<br>• Excess risk of deaths from CHD was 3.03%.<br>• Disutility of RRSO was 0.95 [24] .	• The true HRT compliance in a larger broad-based population was not established.<br>• The range of utility score for RRSO was large.<br>• Complications from RRSO were not included.	• Extensive sensitivity analyses were undertaken<br>• The results were highly sensitive to the disutility of RRSO and HRT compliance.<br>• The impact of CHD is incorporated in the model.
Manchanda, 2015 [15]	• The chance of both BC and OC occurring at the same time was rare and assumed to be close to zero.<br>• 70% of women presenting with OC would be at advanced stage.<br>• Cancer patients 10 years post diagnosis had the same conditional probability of death as the general population.<br>• Costs for identifying/calculating OC-risk were not included.<br>• The OC risk-reduction of RRSO was 94%.<br>• Excess risk of deaths from CHD was 3.03%.<br>• Disutility of RRSO was 0.95 [24] .	• Costs of genetic testing were not included.<br>• The range of utility score for RRSO was large.<br>• Complications from RRSO were not included.	• Extensive sensitivity analyses were undertaken.<br>• The results were sensitive to the disutility of RRSO.
**Women at baseline population OC-risk**			
Cadish, 2017 [16]	• The completion rate of OBS at the time of vaginal hysterectomy was 88%.<br>• 12% of those whose tubes remained in situ required subsequent benign adnexal surgery compared to 4% requiring adnexal surgery after hysterectomy with OBS.<br>• The subsequent benign adnexal surgery performed on a woman who had not previously undergone OBS was either a unilateral or bilateral laparoscopic salpingo-oophorectomy; for those who had previously undergone OBS at the time of hysterectomy, the subsequent surgery was assumed to be either unilateral or bilateral laparoscopic oophorectomy.<br>• The OC risk-reduction was 65% from OBS, and was 21% from hysterectomy.<br>• No data was available regarding the OC risk-reduction from hysterectomy with OBS; it was assumed to be the same as risk-reduction from OBS alone.	• In cases where no prospective evidence was available, retrospective data was used.	• OC risk-reduction from OBS was assumed at 65%. This estimate has a number of limitations and biases. Lower estimates of 28% have recently been reported by the same authors [28].<br>• Uncertainty remains over the effect size of OC risk-reduction from OBS.<br>• The outcome measure was cancer cases/deaths prevented; disutility was not included in the analysis.<br>• Recent data suggest hysterectomy alone may not reduce OC-risk [29].<br>• Results are significantly impacted by uncertainty around effect size of OC risk reduction from OBS.<br>• Disutility from risk-reducing surgery was not considered and can influence results.<br>• Long-term impact of OBS on ovarian function is unknown.<br>• PSA was not undertaken though one-way sensitivity analyses were performed.
Dilley, 2017 [17]	• A probability of prior tubal ligation was assumed for women who underwent hysterectomy without salpingectomy.<br>• Complication rates were not different in groups with or without OBS. Complication rates were 15% for hysterectomy and 1.6% for tubal ligation.<br>• Patients who developed OC and survived were assumed to incur costs only for the initial year with cancer; those who died from OC were assumed to incur costs of the initial year, three additional years, and the final year of life.	• OC incidence and natural history was simplified and deaths unrelated to OC were not taken into consideration, hence introducing bias.<br>• Potential impact of salpingectomy on ovarian function and the	• OC risk-reduction from OBS was assumed at 65%. Salpingectomy needed to reduce OC-risk by 31% at hysterectomy or 54% at sterilization to remain cost-effective. Lower estimates of 28% OC-risk reduction have recently been reported by the same authors [28] .

	• Patients who did not have OC or any other surgical related complications were assumed to be in perfect health with a utility score of 1.<br>• Patients with OC who did not die from OC were assumed to have one year of life at a utility of 0.64, whereas women who died from OC were assumed to have four years of life at this utility.<br>• The OC risk-reduction was 21% from hysterectomy, 28% from tubal ligation, and 65% from OBS.<br>• Disutility from surgical complications was 0.23 for one year.	related morbidity and mortality as well as costs were not included.<br>• OC risk-reduction estimates were dependent on a retrospective study where the actual number of women undergoing OBS was small and salpingectomies were only performed if clinically indicated and not as opportunistic.<br>• The study results were not stratified based on OC histology.	• OBS was assumed to cause no disutility.<br>• Results can be significantly impacted by uncertainty around effect size of OBS and disutility from RRS.<br>• Recent data suggest hysterectomy alone may not reduce OC-risk [29].<br>• Both one-way sensitivity analysis and PSA were undertaken.
Kwon, 2015 [18]	• The majority of OC in the population were high-grade serous carcinomas (70%) and majority of those (70%) arise in the fallopian tube.<br>• The OC risk-reduction was 20% from hysterectomy, 30% from tubal ligation, 50% from OBS, and 90% from BSO.<br>• Premenopausal women who had BSO with hysterectomy were routinely prescribed HRT, but 20% discontinued this by 5 years. Deaths from CHD due to premature menopause from BSO at hysterectomy was modelled.<br>• 1% risk of inadvertent oophorectomy during OBS was assumed.	• There was uncertainty related to the long-term effect on ovarian function and OC risk-reduction from OBS.<br>• Utilities, costs associated with potential morbidity and recurrent OC were not included.<br>• After hysterectomy with BSO, subsequent adnexal cysts, increased risk of osteoporosis and other cancers were not accounted for.<br>• After tubal ligation, possibility for ectopic pregnancy was not considered.	• OC risk-reduction from OBS was assumed at 50%. Lower estimates of 28% OC-risk reduction have recently been reported [28] .<br>• The outcome measure was life-years gained; utility was not included.<br>• Recent data suggest hysterectomy alone may not reduce OC-risk [29].<br>• Results can be significantly impacted by uncertainty around effect size of OBS.<br>• Disutility from risk-reducing surgery was not considered and can influence results.<br>• The long-term impact of salpingectomy on ovarian function is not known.<br>• PSA was not undertaken though one way sensitivity analyses were provided.
Naumann, 2021 [19]	• The OC risk-reduction was 21% from hysterectomy, 33% from tubal ligation, 65% from OBS.<br>• Only the costs of OBS and OC were included.<br>• Only the utility of OC was included.<br>• No disutility from OBS or surgery was included.	• The model did not account for future BSO after hysterectomy.<br>• The future costs of surgery for retained tubes and adnexa were not considered.	• OC risk-reduction from OBS was assumed at 65%. Uncertainty remains over the level of OC risk-reduction from OBS. Lower levels of risk reduction of up to 28% have been reported [28].<br>• The disutility from surgery was not included.<br>• Results can be significantly impacted by uncertainty around effect size of OBS.<br>• Disutility from risk-reducing surgery was not considered and can influence results.<br>• Hysterectomy alone may not reduce OC risk [29].
Subramaniam, 2019 [20]	• If unsuccessful with salpingectomy, tubal ligation would be attempted.<br>• Genetic testing and BSO were not included as a possible option in the model.<br>• There was no possibility of decision-regret; reversal of OBS or tubal ligation were not considered.<br>• OBS increased theatre time by 15 mins.<br>• The procedure-related complication rate was equal between OBS and tubal ligation.	• The true OC-risk reduction associated with OBS and tubal ligation at the time of caesarean was unknown.<br>• Data regarding the complications related to OBS and tubal ligation were limited.	• OC risk-reduction from OBS was assumed at 54%.<br>• The disutility from surgery was not included.<br>• Results can be significantly impacted by uncertainty around effect size of OBS

	• Patients not experiencing unplanned pregnancy or developing OC would have perfect health between the time of cesarean and onset of diagnosis respectively.<br>• The OC risk-reduction was 34% from tubal ligation, and 54% from OBS.	• The estimated cost difference between OBS and tubal ligation was not precise.	• Disutility from risk-reducing surgery was not considered and can influence results.<br>• Higher risk of hemorrhage has been reported with OBS at cesarean section.<br>• Both one way and PSA were undertaken.
Tai, 2018 [21]	• The OC risk-reduction was 50% from OBS.<br>• For women who had an ectopic pregnancy after tubal coagulation/clips, unilateral salpingectomy would be performed as a standard treatment with OC-risk reduction reduced by half.<br>• For health states with durations of <1 year (ectopic, cancer states, and postoperative recovery states), QALYs were adjusted according to the proportion of the year women were in those health states.	• The OC pathway was simplified.<br>• Supportive care costs for palliative chemotherapy for recurrent disease were not included.	• OC risk-reduction from OBS was assumed at 50%.<br>• Uncertainty remains over the OC risk-reduction from OBS.<br>• Disutility from risk-reducing surgery was not considered and can influence results.
Venkatesh, 2019 [22]	• OBS and tubal ligation could be performed in 100% of caesarean sections, and the success rate for completing both types of sterilization was assumed to be 100%.<br>• The OC risk-reduction was 34% from tubal ligation, and 64% from OBS.<br>• Given the paucity of data on OBS with caesarean delivery, data on the risks/benefits were largely derived from OBS with hysterectomy and permanent sterilization.<br>• The perioperative complication rate (including the background complication rate for cesarean section) was 8.3% for OBS and 7.6% for tubal ligation.<br>• Disutility of surgical complications was 0.23 for one year.	• The impact of peripartum OBS on ovarian function and related morbidity was not included.<br>• The study results were not stratified based on OC histology.<br>• Patient decision-regret following sterilization was not included.<br>• Other non-cancer benefits of OBS compared to tubal ligation (apart from contraception efficacy), including elimination of future tubal pathology, were not included.	• OC risk-reduction from OBS was assumed at 64%.<br>• Uncertainty remains over the OC risk-reduction from OBS.<br>• The disutility from surgery was not included and can impact results.

BC, breast cancer; BSO, bilateral salpingo-oophorectomy; CHD; coronary heart disease; CRC, colorectal cancer; DO, delayed oophorectomy; EC, endometrial cancer; ES, early salpingectomy; FH, family history; HER2, human epidermal growth factor receptor 2; HR, hormone receptor; HRT, hormone replacement treatment; LS, Lynch syndrome; MRI, magnetic resonance imaging; OBS, opportunistic bilateral salpingectomy; OC, ovarian cancer; PARP-i, poly ADP ribose polymerase inhibitor; PSA, probabilistic sensitivity analysis; PV, pathogenic variant; QALY, quality-adjusted life-years; RRES, risk-reducing early salpingectomy; RRESDO, risk-reducing early salpingectomy with delayed oophorectomy; RRM, risk-reducing mastectomy; RRS, risk-reducing surgery; RRSO, risk-reducing salpingo-oophorectomy; TNBC, triple-negative breast cancer; USS, ultrasound scan.
*refers to limitations reported by each study. #refers to appraisal by authors of this review.

Reference

1. Anderson, K.; Jacobson, J.S.; Heitjan, D.F.; Zivin, J.G.; Hershman, D.; Neugut, A.I.; Grann, V.R. Cost-effectiveness of preventive strategies for women with a BRCA1 or a BRCA2 mutation. *Annals of Internal Medicine* **2006**, *144*, 397-406.
2. Bommer, C.; Lupatsch, J.; Bürki, N.; Schwenkglenks, M. Cost-utility analysis of risk-reducing strategies to prevent breast and ovarian cancer in BRCA-mutation carriers in Switzerland. *Eur J Health Econ* **2022**, *23*, 807-821, doi:10.1007/s10198-021-01396-9.
3. Grann, V.R.; Panageas, K.S.; Whang, W.; Antman, K.H.; Neugut, A.I. Decision analysis of prophylactic mastectomy and oophorectomy in BRCA1-positive or BRCA2-positive patients. *Journal of Clinical Oncology* **1998**, *16*, 979-985.
4. Grann, V.R.; Patel, P.R.; Jacobson, J.S.; Warner, E.; Heitjan, D.F.; Ashby-Thompson, M.; Hershman, D.L.; Neugut, A.I. Comparative effectiveness of screening and prevention strategies among BRCA1/2-affected mutation carriers. *Breast Cancer Research & Treatment* **2011**, *125*, 837-847.

5. Kwon, J.S.; Tinker, A.; Pansegrau, G.; McAlpine, J.; Housty, M.; McCullum, M.; Gilks, C.B. Prophylactic salpingectomy and delayed oophorectomy as an alternative for BRCA mutation carriers. *Obstet Gynecol* **2013**, *121*, 14-24, doi:http://10.1097/AOG.0b013e3182783c2f
10.1097/aog.0b013e3182783c2f.
6. Muller, D.; Danner, M.; Rhiem, K.; Stollenwerk, B.; Engel, C.; Rasche, L.; Borsi, L.; Schmutzler, R.; Stock, S. Cost-effectiveness of different strategies to prevent breast and ovarian cancer in German women with a BRCA 1 or 2 mutation. *European Journal of Health Economics* **2018**, *19*, 341-353.
7. Norum, J.; Hagen, A.I.; Maehle, L.; Apold, J.; Burn, J.; Møller, P. Prophylactic bilateral salpingo-oophorectomy (PBSO) with or without prophylactic bilateral mastectomy (PBM) or no intervention in BRCA1 mutation carriers: a cost-effectiveness analysis. *Eur J Cancer* **2008**, *44*, 963-971, doi:10.1016/j.ejca.2008.02.025.
8. Petelin, L.; Hossack, L.; Shanahan, M.; Mitchell, G.; Liew, D.; James, P.A.; Trainer, A.H. Cost-effectiveness of long-term clinical management of BRCA pathogenic variant carriers. *Genetics in Medicine* **2020**, *22*, 831-839.
9. Yamauchi, H.; Nakagawa, C.; Kobayashi, M.; Kobayashi, Y.; Mano, T.; Nakamura, S.; Arai, M. Cost-effectiveness of surveillance and prevention strategies in BRCA1/2 mutation carriers. *Breast Cancer* **2018**, *25*, 141-150.
10. Gamble, C.; Havrilesky, L.J.; Myers, E.R.; Chino, J.P.; Hollenbeck, S.; Plichta, J.K.; Kelly Marcom, P.; Shelley Hwang, E.; Kauff, N.D.; Greenup, R.A. Cost Effectiveness of Risk-Reducing Mastectomy versus Surveillance in BRCA Mutation Carriers with a History of Ovarian Cancer. *Annals of Surgical Oncology* **2017**, *24*, 3116-3123.
11. Kwon, J.S.; Sun, C.C.; Peterson, S.K.; White, K.G.; Daniels, M.S.; Boyd-Rogers, S.G.; Lu, K.H. Cost-effectiveness analysis of prevention strategies for gynecologic cancers in Lynch syndrome. *Cancer* **2008**, *113*, 326-335.
12. Wright, J.D.; Silver, E.R.; Tan, S.X.; Hur, C.; Kastrinos, F. Cost-effectiveness Analysis of Genotype-Specific Surveillance and Preventive Strategies for Gynecologic Cancers Among Women With Lynch Syndrome. *JAMA Netw Open* **2021**, *4*, e2123616, doi:10.1001/jamanetworkopen.2021.23616.
13. Yang, K.Y.; Caughey, A.B.; Little, S.E.; Cheung, M.K.; Chen, L.M. A cost-effectiveness analysis of prophylactic surgery versus gynecologic surveillance for women from hereditary non-polyposis colorectal cancer (HNPCC) Families. *Familial cancer* **2011**, *10*, 535-543.
14. Manchanda, R.; Legood, R.; Antoniou, A.C.; Gordeev, V.S.; Menon, U. Specifying the ovarian cancer risk threshold of 'premenopausal risk-reducing salpingo-oophorectomy' for ovarian cancer prevention: a cost-effectiveness analysis. *J Med Genet* **2016**, *53*, 591-599, doi:10.1136/jmedgenet-2016-103800.
15. Manchanda, R.; Legood, R.; Pearce, L.; Menon, U. Defining the risk threshold for risk reducing salpingo-oophorectomy for ovarian cancer prevention in low risk postmenopausal women. *Gynecol Oncol* **2015**, *139*, 487-494, doi:10.1016/j.ygyno.2015.10.001.
16. Cadish, L.A.; Shepherd, J.P.; Barber, E.L.; Ridgeway, B. Risks and benefits of opportunistic salpingectomy during vaginal hysterectomy: a decision analysis. *Am J Obstet Gynecol* **2017**, *217*, 603.e601-603.e606, doi:10.1016/j.ajog.2017.06.007.
17. Dilley, S.E.; Havrilesky, L.J.; Bakkum-Gamez, J.; Cohn, D.E.; Michael Straughn, J., Jr.; Caughey, A.B.; Rodriguez, M.I. Cost-effectiveness of opportunistic salpingectomy for ovarian cancer prevention. *Gynecologic Oncology* **2017**, *146*, 373-379.
18. Kwon, J.S.; McAlpine, J.N.; Hanley, G.E.; Finlayson, S.J.; Cohen, T.; Miller, D.M.; Gilks, C.B.; Huntsman, D.G. Costs and benefits of opportunistic salpingectomy as an ovarian cancer prevention strategy. *Obstetrics & Gynecology* **2015**, *125*, 338-345.
19. Naumann, R.W.; Hughes, B.N.; Brown, J.; Drury, L.K.; Herzog, T.J. The impact of opportunistic salpingectomy on ovarian cancer mortality and healthcare costs: a call for universal insurance coverage. *Am J Obstet Gynecol* **2021**, *225*, 397.e391-397.e396, doi:10.1016/j.ajog.2021.03.032.
20. Subramaniam, A.; Einerson, B.D.; Blanchard, C.T.; Erickson, B.K.; Szychowski, J.; Leath, C.A., 3rd; Biggio, J.R.; Huh, W.K. The cost-effectiveness of opportunistic salpingectomy versus standard tubal ligation at the time of cesarean delivery for ovarian cancer risk reduction. *Gynecol Oncol* **2019**, *152*, 127-132, doi:10.1016/j.ygyno.2018.11.009.
21. Tai, R.W.M.; Choi, S.K.Y.; Coyte, P.C. The Cost-Effectiveness of Salpingectomies for Family Planning in the Prevention of Ovarian Cancer. *J Obstet Gynaecol Can* **2018**, *40*, 317-327, doi:10.1016/j.jogc.2017.06.038.
22. Venkatesh, K.K.; Clark, L.H.; Stamilio, D.M. Cost-effectiveness of opportunistic salpingectomy vs tubal ligation at the time of cesarean delivery. *Am J Obstet Gynecol* **2019**, *220*, 106.e101-106.e110, doi:10.1016/j.ajog.2018.08.032.

23. Grann, V.R.; Jacobson, J.S.; Sundararajan, V.; Albert, S.M.; Troxel, A.B.; Neugut, A.I. The quality of life associated with prophylactic treatments for women with BRCA1/2 mutations. *Cancer J Sci Am* **1999**, *5*, 283-292.
24. Grann, V.R.; Patel, P.; Bharthuar, A.; Jacobson, J.S.; Warner, E.; Anderson, K.; Warner, E.; Tsai, W.Y.; Hill, K.A.; Neugut, A.I.; et al. Breast cancer-related preferences among women with and without BRCA mutations. *Breast Cancer Res Treat* **2010**, *119*, 177-184, doi:10.1007/s10549-009-0373-6.
25. Menon, U.; Gentry-Maharaj, A.; Burnell, M.; Singh, N.; Ryan, A.; Karpinskyj, C.; Carlino, G.; Taylor, J.; Massingham, S.K.; Raikou, M.; et al. Ovarian cancer population screening and mortality after long-term follow-up in the UK Collaborative Trial of Ovarian Cancer Screening (UKCTOCS): a randomised controlled trial. *Lancet* **2021**, *397*, 2182-2193, doi:10.1016/S0140-6736(21)00731-5.
26. Gaba, F.; Blyuss, O.; Chandrasekaran, D.; Osman, M.; Goyal, S.; Gan, C.; Izatt, L.; Tripathi, V.; Esteban, I.; McNicol, L.; et al. Attitudes towards risk-reducing early salpingectomy with delayed oophorectomy for ovarian cancer prevention: a cohort study. *BJOG* **2021**, *128*, 714-726, doi:10.1111/1471-0528.16424.
27. Gaba, F.; Manchanda, R. Systematic review of acceptability, cardiovascular, neurological, bone health and HRT outcomes following risk reducing surgery in BRCA carriers. *Best Pract Res Clin Obstet Gynaecol* **2020**, *65*, 46-65, doi:10.1016/j.bpobgyn.2020.01.006.
28. Falconer, H.; Yin, L.; Salehi, S.; Altman, D. Association between pelvic inflammatory disease and subsequent salpingectomy on the risk for ovarian cancer. *Eur J Cancer* **2021**, *145*, 38-43, doi:10.1016/j.ejca.2020.11.046.
29. Taylor, J.A.; Burnell, M.; Ryan, A.; Karpinskyj, C.; Kalsi, J.K.; Taylor, H.; Apostolidou, S.; Sharma, A.; Manchanda, R.; Woolas, R.; et al. Association of hysterectomy and invasive epithelial ovarian and tubal cancer: a cohort study within UKCTOCS. *Bjog* **2022**, *129*, 110-118, doi:10.1111/1471-0528.16943.
